# Dual‐Target ROS‐Driven Spatiotemporal Senolysis for Vascular Repair and Immune Microenvironment Reprogramming in the Treatment of Ocular Fundus Neovascularization

**DOI:** 10.1002/advs.202523495

**Published:** 2026-02-16

**Authors:** Yali Zhou, Tianxing Chen, Peiyu Liu, Kangjia Lv, Yifan Wang, Xiaoqian Wang, Junwei Fang, Chong Chen, Zhaoyang Wang, Fang Wei, Xun Xu

**Affiliations:** ^1^ Department of Ophthalmology Shanghai General Hospital Shanghai JiaoTong University School of Medicine National Clinical Research Center for Eye Diseases Shanghai Clinical Research Center for Eye Diseases Shanghai Key Clinical Specialty Shanghai Key Laboratory of Ocular Fundus Diseases Shanghai Engineering Center for Visual Science and Photomedicine Shanghai Engineering Center for Precise Diagnosis and Treatment of Eye Diseases Shanghai China; ^2^ Beijing Institute of Ophthalmology Beijing Tongren Hospital Capital Medical University Beijing China; ^3^ Beijing Tongren Eye Center Beijing Tongren Hospital Capital Medical University Beijing China

**Keywords:** immune microenvironment reprogramming, ocular fundus neovascularization, senolysis, single‐cell RNA sequencing, vascular repair

## Abstract

Ocular fundus neovascularization (OFN) is a leading cause of irreversible vision loss. Conventional antivascular endothelial growth factor (anti‐VEGF) therapies indiscriminately suppress pathological and reparative angiogenesis and fail to correct the senescence‐ and inflammation‐driven microenvironment that sustains disease progression. Senescent endothelial cells (ECs) form the structural scaffold of pathological vessels, while neighboring senescent microglia exacerbate inflammatory signaling, together deteriorating the reactive oxygen species (ROS)‐rich vascular‐immune microenvironment. Here, we develop an injectable ROS‐responsive senolytic hydrogel (PCC1/PHCF‐Gel) that enables lesion‐activated, sustained intraocular release of procyanidin C1 (PCC1), overcoming rapid clearance, oxidative degradation, and poor lesion retention associated with free PCC1. In oxygen‐induced retinopathy and choroidal neovascularization models, PCC1/PHCF‐Gel markedly reduces retinal senescence, suppresses pathological neovascularization, and restores neuroretinal function, outperforming symptom‐directed therapies anti‐VEGF therapy. Single‐cell RNA sequencing reveals selective elimination of two pathogenic senescent cell subpopulations—CXCR4^+^ ECs and IFITM3^+^ microglia—thereby disrupting the reinforcing cycle of vascular and immune senescence and promoting reparative vascular regeneration. These findings establish a multifunctional, spatiotemporally controlled therapeutic paradigm and highlight PCC1/PHCF‐Gel as a promising translational strategy for the precision treatment of OFN.

## Graphical abstract

We developed a senolytic approach using procyanidin C1 (PCC1)‐loaded platform, a clinically established and safe hyaluronic acid‐based scaffold, to selectively eliminate senescent endothelial and microglial cells. This dual‐targeting strategy significantly suppresses pathological neovascularization and promotes vascular repair, presenting a safe and translational therapeutic paradigm for ocular fundus neovascularization (OFN).

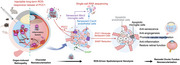

## 1 Introduction

The eye has emerged as a pivotal organ in mediating rapid physiological responses to environmental threats and consequently receives a high level of metabolic support, primarily delivered through the retinal and choroidal vascular networks [1]. Disruption of these vascular networks produces localized ischemia, which in turn induces ocular fundus neovascularization (OFN) that ultimately impairs the transmission of visual signals [2]. The breakdown of these vascular networks is a major cause of visual impairment, particularly in industrialized countries. OFN manifests at all ages, as retinopathy of prematurity in infants, diabetic retinopathy (DR) in working‐age adults, and age‐related macular degeneration (AMD) in the elderly [3, 4, 5]. Retinopathy of prematurity affects 14,000–16, 000 infants annually in the United States, with 90% resolving spontaneously, yet of the ~1500 requiring treatment, one‐third progress to legal blindness [6]. Among adults of working age, DR affects more than 100 million people globally [7]. AMD is anticipated to affect nearly 300 million elderly individuals worldwide by 2040, with approximately 30% developing the neovascular subtype (nAMD), which is defined by choroidal neovascularization (CNV) [8].

Current standard care relies on repeated intravitreal anti‐vascular endothelial growth factor (anti‐VEGF) injections, which indiscriminately block both pathological and physiological angiogenesis by suppressing endothelial cell (EC) responses to VEGF signals derived from hypoxia‐activated neurons and glial cells [9, 10]. Therefore, persistent VEGF neutralization fails to resolve retinal ischemia and hypoxia, thereby accelerating photoreceptor atrophy and contributing to recurrence in many patients [11]. Alternatively, laser photocoagulation ablates ischemic retina to reduce the production of angiogenic and inflammatory factors, but irreversibly and permanently destroys peripheral neuroretinal tissue as a cost [12]. Crucially, both approaches disregard that certain forms of neoangiogenesis are reparative, which can restore functional vasculature to ischemic tissue and mitigate the hypoxic stress driving disease progression [11, 13, 14]. Therefore, the development of novel therapies that can selectively target pathological vessels while preserving reparative vascular function, thereby remodeling the pathological microenvironment, is essential for treating OFN.

Recent studies have shown that pathological vessels exhibit disorganized architecture, increased permeability, and enrichment of senescent cells. In particular, senescent ECs expressing p16^INK4a^ have been reported to accumulate in ocular tissues from patients [15, 16]. These cells maintain an anti‐apoptotic state and metabolic activity despite permanent cell cycle arrest, secreting a broad secretome of inflammatory factors through the senescence‐associated secretory phenotype (SASP) [17]. This state critically impairs the self‐repair of ECs while recruiting immune cells and triggering microglial activation with autocrine senescence [18, 19]. Moreover, senescent ECs serve as structural foundations for the pathological vessels, while the local coexistence of senescent microglia further amplifies inflammatory signaling, thereby sustaining the reactive oxygen species (ROS)‐enriched inflammatory‐angiogenic microenvironment [20]. Based on the distinct characteristics of cellular senescence and apoptosis resistance, which present a therapeutic challenge but also reveal a point of vulnerability in the disease mechanism, a novel therapeutic strategy called senolysis has been created.

Recently, senolytic therapy has emerged as a promising paradigm for treating OFN, yet only a few research teams have explored this treatment strategy and screened a limited number of drugs. Preclinical studies in models of DR and nAMD have reported promising results, with senolytic clearance effectively restoring vascular integrity and mitigating inflammation [15, 19, 21]. Moreover, a phase 1 clinical trial (NCT04537884) evaluating BCL‐xL inhibition with UBX1325 (foselutoclax) in anti‐VEGF‐refractory patients demonstrated clinical activity in diabetic macular edema [22]. However, inhibition of BCL‐xL, a key regulator of cell survival and apoptosis, carries the risk of unintended damage to retinal neurons. In addition, current studies remain largely restricted to the clearance of ECs, which alone cannot effectively disrupt the local vascular‐inflammatory feedback loop between ECs and microglia [15, 22]. Therefore, it is essential to develop safer and dual‐target anti‐senescent agents that can eliminate both ECs and microglia simultaneously.

Recent studies demonstrate that procyanidin C1 (PCC1), a natural trimeric epicatechin flavonoid with a favorable safety profile, exhibits potent senolytic activity across diverse cell types [23]. In senescent PSC27 prostate stromal cells, PCC1 induces apoptosis, which involves the upregulation of the pro‐apoptotic BH3‐only proteins NOXA and PUMA [23]. Furthermore, PCC1 ablates senescent human cell models, including fetal lung fibroblasts (WI38), primary human umbilical vein ECs, and human mesenchymal stem cells [23]. PCC1 also reduces cellular senescence in retinal cell models in vitro, including 661W photoreceptor cells and ARPE‐19 retinal pigment epithelial cells [24]. Notably, PCC1 can reverse the elevated proportion of microglia observed in naturally aged retinas [24]. Moreover, it can alleviate renal fibrosis by eliminating senescent renal tubular epithelial cells and can reverse pulmonary fibrosis by targeting senescent myofibroblasts [25, 26]. These findings demonstrate PCC1's broad senolytic activity across multiple tissues and cell types. This suggests that PCC1 has great potential to simultaneously target and clear senescent ECs and microglia in OFN, a strategy that remains unexplored.

**FIGURE 1:**
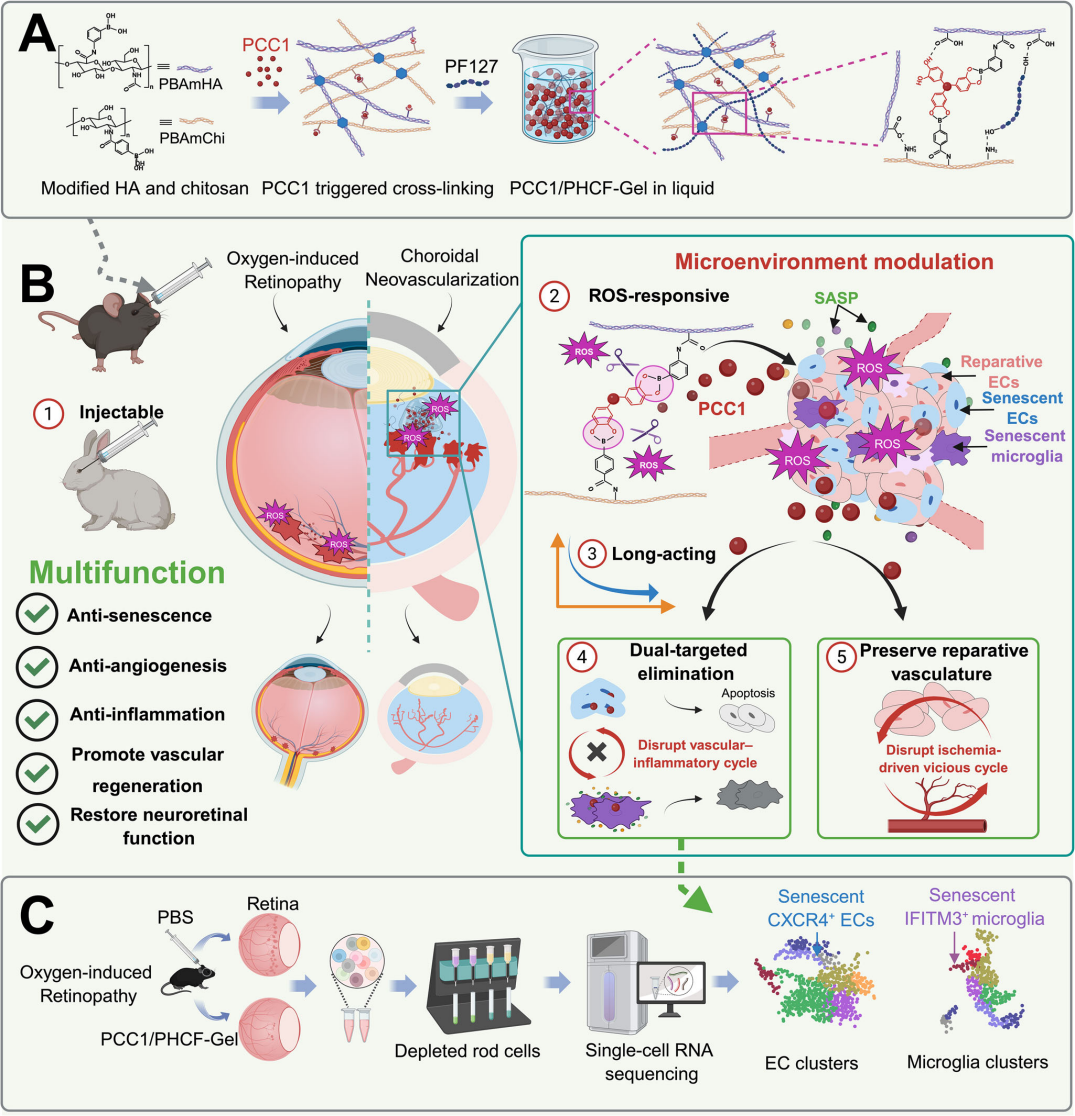
Schematic illustration of PCC1/PHCF‐Gel for sustained release and multifunctional treatment of OFN. (A) Preparation of PCC1/PHCF‐Gel. (B) In the OIR and CNV models, the ROS‐responsive hydrogel enabled sustained PCC1 release to selectively eliminate senescent ECs and microglia, thereby disrupting the vascular‐inflammatory vicious cycle, preserving reparative vasculature and, in turn, disrupting the ischemia‐driven vicious cycle, while delivering synergistic anti‐angiogenic, anti‐inflammatory, pro‐regenerative, and neuroprotective benefits. (C) scRNA‐seq analysis identified the specific cell subpopulations eliminated by the gel's senolytic effects.

Based on previous literature, ameliorating the cellular senescence microenvironment through intravitreal delivery of PCC1 holds great potential for promoting healthy vascular regeneration in OFN. However, achieving stable and sustained intravitreal administration of PCC1 remains a significant challenge. Rapid clearance through the vitreous‐aqueous circulation limits its ocular retention, while poor chemical stability arising from oxidation‐prone catechol groups leads to degradation in the ROS‐rich pathological microenvironment [27]. In addition, its non‐specific distribution restricts accumulation at lesion sites, collectively undermining its therapeutic efficacy. To overcome these issues, we engineered an injectable, multifunctional, ROS‐responsive hydrogel that facilitates continuous and targeted release of PCC1 over 1 month and achieves a high therapeutic efficacy in OFN. The network was initially formed using PCC1 as a dynamic crosslinker to connect boronic acid‐modified hyaluronic acid (PBAmHA) and chitosan (PBAmChi) via boronate ester bonds, thus enhancing drug loading while forming a ROS‐sensitive controlled‐release unit. The network was further modified with Pluronic F127 (PF127), which self‐assembles into a thermoresponsive gel through hydrogen bonding, facilitating in situ solidification after injection (Figure 1A). Then, across in vitro senescent‐cellassays and two in vivo models of oxygen‐induced retinopathy (OIR) and laser‐induced CNV, the PCC1/PHCF‐Gel demonstrated superior anti‐senescence, anti‐angiogenic efficacy and improved neoretinal function compared with free PCC1 and conventional anti‐VEGF therapy. Mechanistically, PCC1/PHCF‐Gel coupled ROS‐triggered, lesion‐localized sustained PCC1 release with apoptosis‐mediated senolysis to disrupt the EC‐microglia vascular‐inflammatory feedback loop and thereby shifted the endothelial compartment toward a more reparative state (Figure 1B). Furthermore, bulk RNA sequencing(RNA‐seq) shows a significant reduction in retinal senescent burden, while single‐cell RNA sequencing (scRNA‐seq) identifies CXCR4^+^ senescent ECs and IFITM3^+^ senescence‐associated microglia as selectively eliminated subpopulations, which were enriched in pathological lesions and minimally detected in healthy retina (Figure 1C). Together, these data support PCC1/PHCF‐Gel as a multifunctional, spatiotemporally controlled senolytic platform that remodels the diseased microenvironment, breaks self‐amplifying pathological angiogenic circuits, and enables durable vascular repair, highlighting its translational potential for precision therapy of OFN.

## 2 Results and Discussion

### 2.1 Characterization and Gelation of PCC1/PHCF‐Gel

**FIGURE 2:**
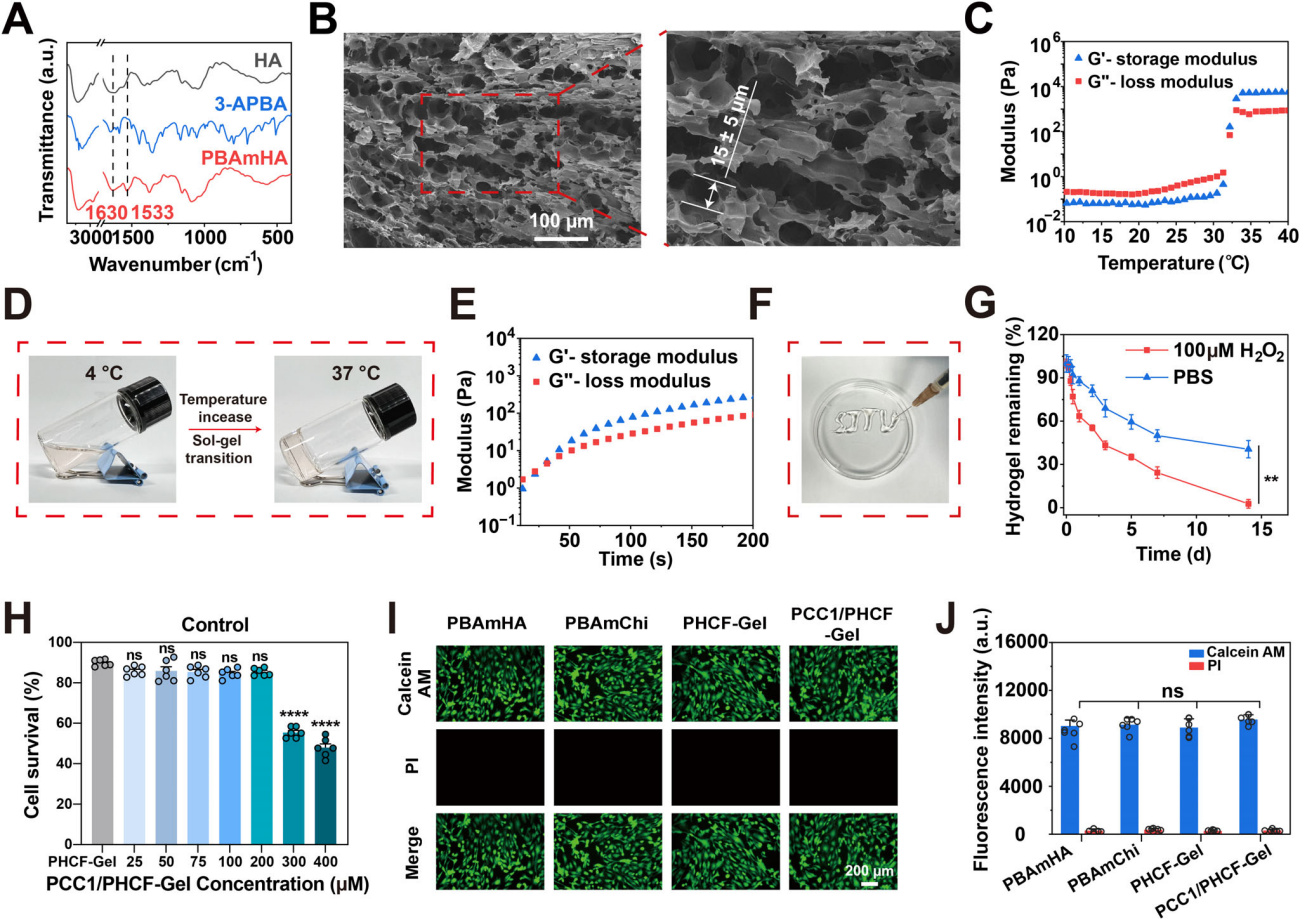
Characterization and gelation of PCC1/PHCF‐Gel. (A) FTIR analysis of HA, 3‐APBA, and PBAmHA. (B) A scanning electron microscopy image of PCC1/PHCF‐Gel (PCC1/PHCF‐Gel‐MC). The mean pore diameter was 15 µm (±5 µm). Scale bar: 100 µm. (C) Temperature‐dependent storage modulus (G′) and loss modulus (G′′) of the PCC1/PHCF‐Gel with a measured gelation temperature of 30.71 °C. (D) Sol‐gel transition of the PCC1/PHCF‐Gel. (E) Temperature‐dependent G′ and G′′ of the PCC1/PHCF‐Gel‐MC hydrogel at the Tgel shown in (C). The gelation time was estimated to be 22 s. (F) The letters "SJTU" pattern were printed using a syringe and the PCC1/PHCF‐Gel as a bioink. (G) Degradation of the PCC1/PHCF‐Gel in PBS or 100 µM H_2_O_2_ at 37 °C (*n* = 3). H) Survival of control (proliferating) HRMECs under increasing PCC1/PHCF‐Gel concentrations (*n* = 6). (I) Live/dead staining of HRMECs cultured with PBAmHA, PBAmChi, PHCF‐Gel, and PCC1/PHCF‐Gel, with calcein AM and PI as indicators. Green fluorescence represents living cells. Scale bar: 200 µm. (J) Quantitative analysis of Live/dead staining intensity using ImageJ (*n* = 6). The *p*‐values were calculated using two‐way analysis of variance (ANOVA) with Bonferroni's test for G), and one‐way ANOVA followed by Tukey's post hoc test for (H and J). Data are presented as the mean ± SEM. ^**^ *p* < 0.01, ^****^ *p* 0.0001; ns, not significance.

To prepare PBAmHA and PBAmChi, hyaluronic acid (HA) and chitosan were modified through amidation catalyzed by 4‐(4,6‐dimethoxy‐1,3,5‐triazin‐2‐yl)‐4‐methylmorpholinium chloride. As shown in Figure 2A, compared with HA and 3‐aminophenylboronic acid (3‐APBA), new peaks appeared in the fourier transform infrared spectroscopy (FTIR) spectrum of PBAmHA at 1630 cm^−1^ (amide I, due to C═O stretching vibration) and at 1533 cm^−1^ (amide II, corresponding to N‐H bending), indicating that phenylboronic acid was successfully grafted onto the HA chains. Similar FTIR results were obtained for PBAmChi (Figure S1, Supporting Information), as evidenced by the distinct spectral changes relative to chitosan and 4‐carboxyphenylboronic acid (4‐CPBA), with new peaks at 1632 and 1600 cm^−1^ attributed to amide I and amide II, respectively. The phenylboronic acid modification was further confirmed by proton nuclear magnetic resonance (^1^H NMR). Characteristic chemical shifts at 7.46, 7.58, 7.64, and 7.86 ppm for PBAmHA (Figure S2) and at 7.77, 7.79, 7.85, and 7.86 ppm for PBAmChi (Figure S3) correspond to phenylboronic acid protons, confirming the successful conjugation to both HA and chitosan backbones. The degree of substitution was calculated as 33.55% for PBAmHA and 5.37% for PBAmChi.

PBAmHA and PBAmChi were further crosslinked with PCC1 to form a hydrogel network, PCC1/PHCF, with a weight ratio of PBAmHA to PBAmChi of 2:1. The mixture was purged with nitrogen to eliminate oxygen and stirred in the dark for 12 h at 4 °C. Next, the hydrogel network was modified with 15% PF127 to obtain PF127‐containing gel formulations. To evaluate the influence of PCC1 on the hydrogel properties, a series of PCC1/PHCF hydrogels was generated with a weight ratio of PCC1 to the total polymer of 0.1%, 0.7%, and 1.5%, designated as PCC1/PHCF‐Gel‐LC, PCC1/PHCF‐Gel‐MC, and PCC1/PHCF‐Gel‐HC, respectively. The internal microstructure of the hydrogels was visualized using scanning electron microscopy (Figure 2B and Figure S4). A hydrogel without PCC1 (PHCF‐Gel) was also evaluated as a control. With increasing PCC1‐to‐polymer ratio, the average pore size decreased significantly from 28 µm in PHCF‐Gel, 18 µm in PCC1/PHCF‐Gel‐LC, and 15 µm in PCC1/PHCF‐Gel‐MC, to 11 µm in PCC1/PHCF‐Gel‐HC, indicating a progressive increase in crosslinking density.

The structural features and thermoresponsive behavior of the PCC1/PHCF hydrogel under physiological conditions were then investigated using a rheological analysis. The temperature‐dependent storage modulus (G′) and loss modulus (G′′) of PCC1/PHCF‐Gel‐MC are shown in Figure 2C. Below the gelation temperature (T_gel_), G′ (0.06 Pa) was less than G′′ (0.18 Pa), indicating a liquid‐like behavior. A gelation temperature of 30.71 °C was determined at the intersection of G′ and G′′. Upon further heating, G′ increased sharply to 5633 Pa, surpassing G′′ (784 Pa), indicating a sol‐gel transition and the formation of a solid‐like hydrogel. The T_gel_ values of PF127, PHCF‐Gel, and PCC1/PHCF‐Gel‐LC were 24.95, 24.92, and 25.80 °C, respectively, with corresponding equilibrium G′ values of 4851, 5181, and 5289 Pa (Figure S5A–C). The PCC1/PHCF‐Gel‐MC hydrogel exhibited the highest T_gel_ value and the greatest solid‐state strength, which was attributed to increased boronate ester formation that increased the crosslinking density. The PCC1/PHCF‐Gel‐MC hydrogel appeared as a transparent liquid at 4°C and underwent a sol‐gel transition as the temperature rose to physiological body temperature (37°C) (Figure 2D), indicating suitability for injection. In contrast, the PCC1/PHCF‐Gel‐HC hydrogel (Figure S5D) exhibited a consistently higher G′ than G′′ from 10 to 40 °C, indicating a consistently solid state and the absence of a sol‐gel transition, likely due to excessive crosslinking, which rendered it unsuitable for injection.

Time sweep experiments were performed at T_gel_ to determine the gelation time. As shown in Figure 2E, the gelation time of PCC1/PHCF‐Gel‐MC was 22 s, compared with 66 s for PF127 (Figure S6A) and 46 s for PHCF‐Gel (Figure S6B). The faster gelation of PCC1/PHCF‐Gel‐MC was attributed to its higher PCC1 concentration, which accelerated boronate ester formation. With its appropriate T_gel_, mechanical strength, and fast thermoresponsive gelation, the PCC1/PHCF‐Gel‐MC hydrogel was used as a bioink in a syringe to print the letters “SJTU” (Figure 2F). In subsequent in vivo rabbit studies, the PCC1/PHCF‐Gel‐MC hydrogel demonstrated excellent injectability and self‐healing. The hydrogel was smoothly injected intravitreally and promptly reformed into a gel in tissue (Videos S1), whereas intravitreal injection of PBS (control) produced a bubble (Video S2). Hydrogel self‐healing is critical for maintaining the structural integrity of encapsulated therapeutic agents and to prevent leakage or sedimentation during administration. Thus, PCC1/PHCF‐Gel‐MC (PCC1/PHCF‐Gel) was selected from the tested hydrogels as the optimal candidate for PCC1 delivery due to its favorable characteristics for both storage and administration.

In addition to the hydrogel injectability, its biodegradability and biosafety are crucial for its future use in biomedical applications. To simulate in vivo degradation, the PCC1/PHCF‐Gel was immersed in PBS with or without H_2_O_2_ at 37 °C, and the remaining mass was recorded over time. As shown in Figure 2G, hydrogel degradation was significantly accelerated in the presence of H_2_O_2_, consistent with the ROS‐mediated cleavage of the boronate ester bonds in the hydrogel. The cytotoxicity of PCC1/PHCF‐Gel was assessed using a cell counting kit‐8 (CCK‐8) assay. As shown in Figure 2H, human retinal microvascular endothelial cells (HRMECs) remained viable when cultured with PCC1/PHCF‐Gel at a PCC1 concentration of up to 200 µM. Furthermore, as indicated by calcein acetoxymethyl ester (AM) and propidium iodide (PI) (Figure 2I,J) fluorescence in the live/dead staining results, HRMECs cultured with PBAmHA, PBAmChi, PHCF‐Gel, and PCC1/PHCF‐Gel displayed negligible red fluorescence, indicating few dead cells, and therefore extremely low toxicity. Figure S7 further confirmed that the cell viability was >95% compared with the controls, indicating good biocompatibility. Chitosan and HA are widely used as biocompatible polymers in drug delivery, and the results here indicated that the introduction of phenylboronic acid groups with subsequent crosslinking did not compromise their cytocompatibility.

### 2.2 In Vitro Senolytic Efficacy of PCC1/PHCF‐Gel Against Senescent HRMECs

To investigate the senolytic activity of PCC1/PHCF‐Gel, a series of in vitro assays were conducted on HRMECs, including senescence‐associated β‐galactosidase (SA‐β‐gal) staining, which labels senescent cells with a blue‐green dye, immunofluorescence (IF) staining showing increased expression of established senescence markers γH2AX and p21, and flow cytometric analysis of apoptosis [28]. We first established a senescent HRMECs model by exposing cells to oxidative stress induced by 150 µM H_2_O_2_ for 24 h, followed by five days of culture (Figures S8 and S9).

**FIGURE 3:**
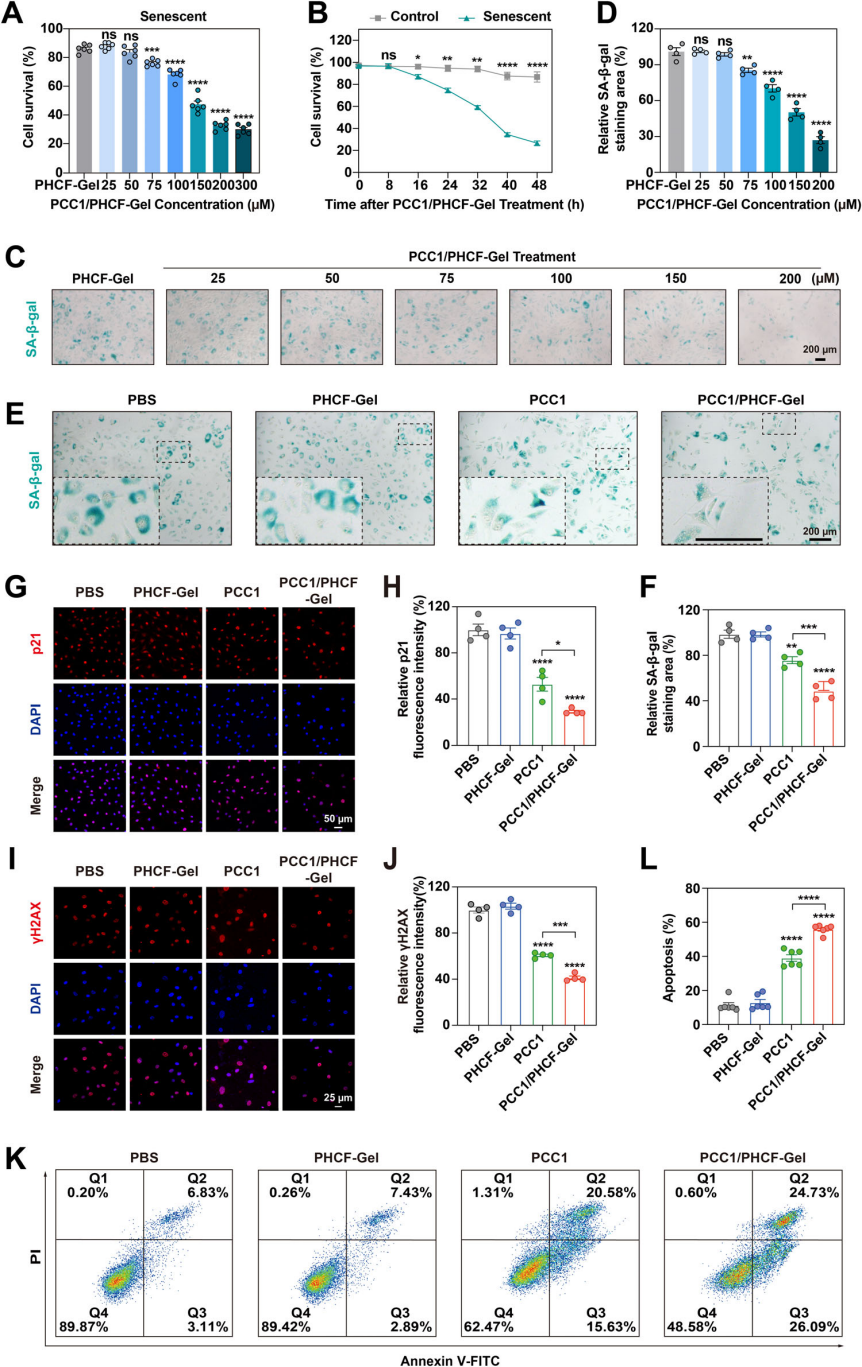
In vitro senolytic efficacy of PCC1/PHCF‐Gel against senescent HRMECs. (A) Survival of H_2_O_2_‐induced senescent HRMECs exposed to PCC1/PHCF‐Gel with increasing concentrations of PCC1 (*n* = 6). (B) Time‐dependent assessment of the viability of control (normal) and senescent HRMECs treated with PCC1/PHCF‐Gel at a concentration of 75 µM (*n *= 3). (C) Representative SA‐β‐gal staining images of senescent HRMECs treated with PHCF‐Gel and varying concentrations of PCC1/PHCF‐Gel. Scale bar: 200 µm. (D) Survival of senescent HRMECs based on SA‐β‐gal staining area (*n* = 4). (E) Representative images depicting SA‐β‐Gal staining in senescent HRMECs after treatment with PBS, PHCF‐Gel, PCC1, and PCC1/PHCF‐Gel. Scale bar: 200 µm. (F) Quantitative analysisSA‐β‐gal staining area (*n* = 4). (G) Confocal images of senescent HRMECs stained after treatment with PBS, PHCF‐Gel, PCC1, and PCC1/PHCF‐Gel. Red and blue colors indicate p21 signals and 4′,6‐diamidino‐2‐phenylindole (DAPI) staining of nuclei, respectively. Scale bar: 50 µm. (H) Quantification of relative p21 fluorescence intensity (*n* = 4). (I) Confocal images showing senescent HRMECs stained for γH2AX (red) and nuclei (DAPI; blue) following treatment with PBS, PHCF‐Gel, PCC1, and PCC1/PHCF‐Gel. Scale bar: 25 µm. (J) Quantification of the relative γH2AX fluorescence intensity (*n* = 4). (K) Flow cytometry‐based apoptosis analysis in senescent HRMECs using Annexin V‐FITC and PI staining. (L) Quantification of apoptotic (Q2 [PI^+^/Annexin V^+^] and Q3 [PI^−^/Annexin V^+^]) cell percentages (*n* = 6). All data are presented as means ± SEM. For data in (A, D, F, H, J, and L), one‐way ANOVA was performed followed by Tukey’s post hoc test. For (B), differences between groups were assessed using an unpaired two‐sided t‐test. ^*^ *p* < 0.05, ^**^ *p* < 0.01, ^***^ *p *< 0.001, and ^****^ *p* < 0.0001; ns, not significance.

Next, we conducted a CCK‐8 assay to evaluate the effects of various concentrations of PCC1/PHCF‐Gel on the viability of senescent HRMECs. The results showed that PCC1/PHCF‐Gel exerted a senolytic effect on senescent HRMECs at a concentration of 75 µM and higher (Figure 3A). Given that PCC1/PHCF‐Gel was shown to cause minimal toxicity of control cells at concentrations of 200 µM and less (Figure 2H), normal HRMECs tolerated much higher concentrations of PCC1/PHCF‐Gel than senescent cells. At a dose of 75 µM, PCC1/PHCF‐Gel effectively lowered the viability of senescent cells without affecting healthy, dividing cells, and provided a practical selectivity window for subsequent studies. This selective cytotoxicity underscored the potential of PCC1/PHCF‐Gel to selectively target senescent ECs while preserving vascular cell viability. We chose 75 µM PCC1/PHCF‐Gel for subsequent mechanistic studies based on its selective efficacy and selectivity window. A time‐course analysis using a CCK‐8 assay revealed that PCC1/PHCF‐Gel began to reduce viability within 16 hours, with a more pronounced effect in senescent HRMECs than in control cells, and plateaued by 48 h (Figure 3B). SA‐β‐gal staining combined with a quantitative analysis confirmed the dose‐dependent, selective clearance of senescent HRMECs by PCC1/PHCF‐Gel (Figure 3C,D).

To further evaluate the effects of PBS, PHCF‐Gel, PCC1, and PCC1/PHCF‐Gel on senescent HRMECs, SA‐β‐gal staining was performed to monitor the change in cell number with treatment. The number of SA‐β‐gal positive cells was significantly reduced by PCC1 and PCC1/PHCF‐Gel compared to the PBS control. A quantitative analysis of the staining area further confirmed that PCC1/PHCF‐Gel was more effective than PCC1 alone, whereas PHCF‐Gel was not significantly different from PBS (Figure 3E,F). IF staining and quantification revealed that PCC1 reduced the proportion of senescent cells (γH2AX^+^/p21^+^), and PCC1/PHCF‐Gel showed an even greater reduction in the senescent cell proportion. In contrast, PHCF‐Gel had no significant effect (Figure 3G–J).

Finally, we used flow cytometry using Annexin V‐FITC/PI dual staining to assess whether senescent HRMECs underwent apoptosis following indicated treatments. The analysis quantified the proportions of apoptotic (Q2 [PI^+^/Annexin V^+^] and Q3 [PI^−^/Annexin V^+^]) cell populations. Both PCC1 and PCC1/PHCF‐Gel promoted apoptosis in senescent HRMECs, with a more pronounced effect with PCC1/PHCF‐Gel (Figure 3K,L). These data indicate that PCC1/PHCF‐Gel can effectively induce apoptosis in senescent cells, supporting its potential for the treatment of OFN.

### 2.3 In Vivo Biocompatibility, ROS‐Responsiveness, and Long‐Term Efficacy of PCC1/PHCF‐Gel

**FIGURE 4:**
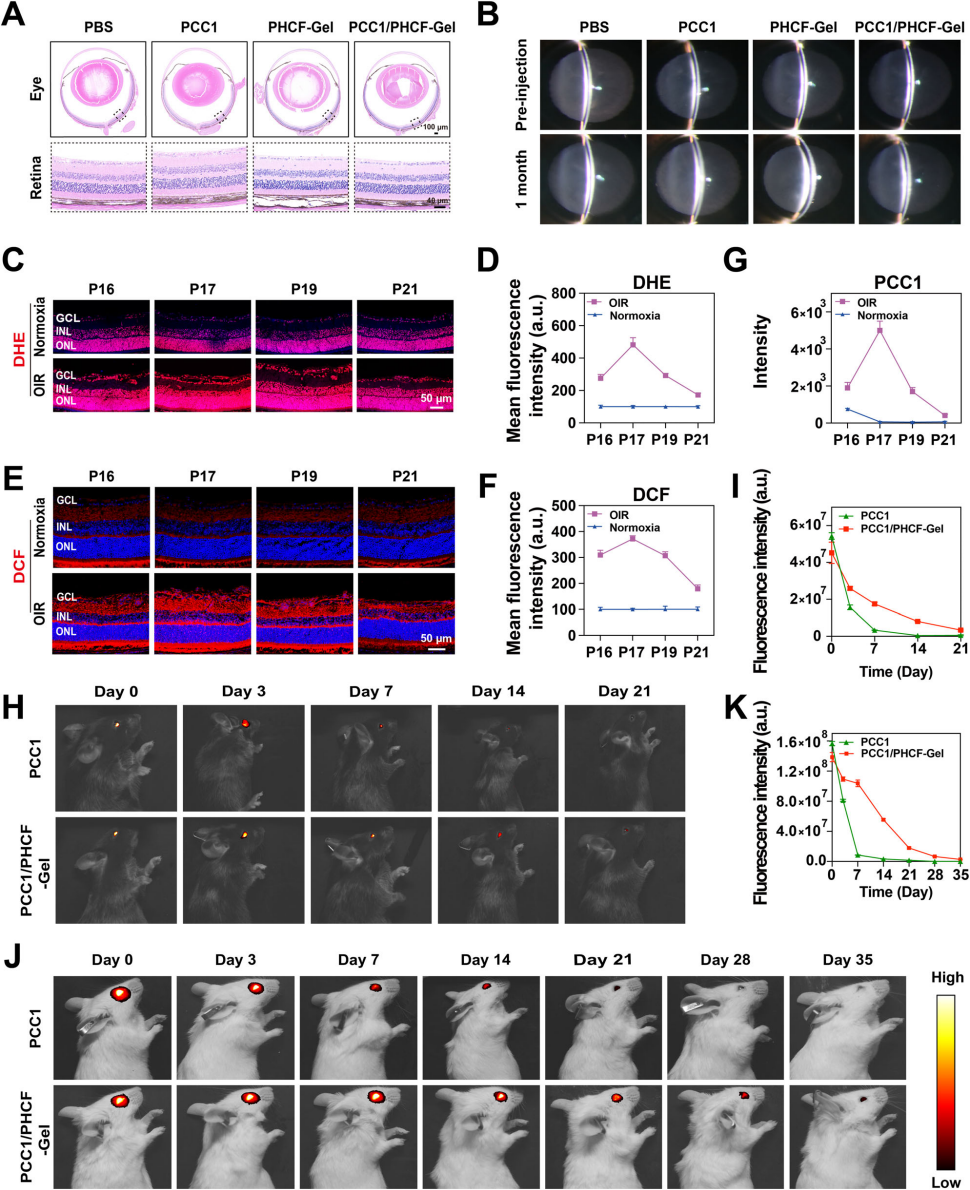
In vivo biocompatibility, ROS‐responsiveness, and long‐term efficacy of PCC1/PHCF‐Gel. (A) Representative images of H&E‐stained sections of ocular tissues from mice that received intravitreal injections of PBS, PCC1, PHCF‐Gel, and PCC1/PHCF‐Gel (*n* = 3). Ocular, Scale bar: 100 µm. Retina, Scale bar: 40 µm. (B) Representative slit‐lamp microscopy images demonstrating preserved anterior segment integrity, with no significant differences in cell/flare scores and transparent corneas and lenses among all treatment groups (PBS, PCC1, PHCF‐Gel, and PCC1/PHCF‐Gel) up to 1 month postinjection (*n* = 3). (C) Confocal micrographs of retinal sagittal sections stained with DHE (red), (E) DCF (red) and nuclei (DAPI, blue) demonstrate the spatiotemporal expression patterns of ROS in the retinas of normoxia and OIR model mice at four critical time points: P16 (proliferation phase), P17 (peak phase), P19 (early regression phase), and P21 (late regression phase). DHE and DCF, Scale bars: 50 µm. (D) Quantitative analysis of ROS levels in frozen retinal sections of normoxia and OIR model mice as detected using DHE (*n* = 4) and (F) DCF staining (*n* = 5). (G) PCC1 levels in the retina were quantitatively analyzed using LC‐QQQ‐MS 1 day (P16), 2 days (P17), 4 days (P19), and 6 days (P21) post‐injection in the OIR model (*n* = 6). (H) IVIS of C57BL/6J mice and (J) BALB/c mice to determine the in vivo retention of PCC1‐Cy5 at different time points after intravitreal injection of either free PCC1‐Cy5 and PCC1/PHCF‐Gel‐Cy5. I) Quantitative analysis of the overall fluorescence intensity of PCC1‐Cy5 and PCC1/PHCF‐Gel‐Cy5 in C57BL/6J mice (*n* = 3) and K) BALB/c mice (*n* = 5). All data are presented as means ± SEM. For data in (D, F and G), two‐way ANOVA was performed. For (I and K), a two‐way mixed‐effects model was used.

The in vivo biocompatibility was tested in wild‐type mice. Following intravitreal administration of PHCF‐Gel or PCC1/PHCF‐Gel, hematoxylin and eosin (H&E) staining revealed no noticeable alterations in ocular tissues, including corneal thickness, iris morphology, or retinal layers, compared with the PBS control (Figure 4A and Figure S10). Representative H&E‐stained sections of major organs (heart, liver, spleen, lung, and kidney) collected on days 7 and 14, and at 1 and 3 months after PCC1/PHCF‐Gel treatment showed no pathological abnormalities (Figure S11). Blood biochemistry and hematological analyses also demonstrated stable profiles at the corresponding time points, all remaining within physiological ranges (Figure S12). Slit‐lamp examinations performed at1 month after intravitreal injection showed that the cornea remained clear and the lens was transparent in all treatment groups (PBS, PCC1, PHCF‐Gel, and PCC1/PHCF‐Gel; Figure 4B). These findings collectively indicate that PCC1/PHCF‐Gel is well tolerated, with no detectable ocular or systemic adverse effects, consistent with previous in vitro safety observations.

To evaluate the responsiveness of PCC1/PHCF‐Gel to pathological changes in the ROS‐rich microenvironment and its capacity for sustained in vivo release of PCC1, we performed dual fluorescence staining using dihydroethidium (DHE) and 2′,7′‐dichlorofluorescein diacetate (DCF) [29]. We systematically analyzed the spatial and temporal distribution of ROS in retinas from normoxic mice and from mice subjected to OIR modeling. Pathological neovascularization emerged around P15, with vessel complexity and number rapidly increasing by P17, at which point dense vascular clusters extended into the vitreous. From P18 to P21, these aberrant vascular complexes underwent regression characterized by progressive vessel pruning and apoptosis [30]. Accordingly, frozen retinal sections were collected at four key time points: P16 (proliferation phase), P17 (peak phase), P19 (early regression), and P21 (late regression) for double‐staining analysis.

Subsequent quantitative analysis showed that, compared with normoxic controls, the OIR group exhibited significantly higher relative fluorescence intensities of DHE and DCF at all four time points. These changes were closely correlated with the pathological progression of neovascularization. The ROS signal intensity peaked at P17, coinciding with maximal pathological vessel density and far exceeding the ROS levels in the control group. During the regression phase (P19 and P21), ROS levels declined markedly in parallel with the gradual regression of the vascular network. These findings demonstrate a strong temporal association between ROS dynamics and pathological angiogenesis in the OIR model, supporting the concept that sustained ROS accumulation contributes to the pathogenesis of retinopathy (Figure 4C‐F).

Next, we evaluated the ROS‐responsive release behavior of the PCC1/PHCF‐Gel delivery system by quantifying retinal PCC1 levels using liquid chromatography‐triple quadrupole tandem mass spectrometry (LC‐QQQ‐MS) [31]. Intravitreal injections were administered to OIR mice at P15, and retinal PCC1 levels were quantified on days 1 (P16), 2 (P17), 4 (P19), and 6 (P21) post‐injection. Retinal PCC1 concentrations exhibited a strong positive correlation with ROS levels at each time point, consistent with the ROS‐responsive, controlled‐release profile of PCC1/PHCF‐Gel and underscoring its potential for lesion‐responsive antioxidant delivery (Figure 4G).

Building on the pharmacokinetic analysis of ROS‐responsive drug release, we next evaluated the intraocular delivery characteristics of PCC1/PHCF‐Gel using in vivo fluorescence imaging (Vieworks IVIS Smart LF system) in two mouse strains with distinct genetic backgrounds, C57BL/6J and BALB/c. Intravitreal injections of either free Cy5‐labeled PCC1 (PCC1‐Cy5) or hydrogel‐encapsulated PCC1‐Cy5 (PCC1‐Cy5@PHCF‐Gel) were administered. In C57BL/6J mice, free PCC1‐Cy5 rapidly diffused from the vitreous cavity, with fluorescence nearly undetectable by day 14, whereas PCC1‐Cy5@PHCF‐Gel produced a sustained signal that persisted for at least 21 days (Figure 4H,I). An even stronger effect was observed in BALB/c mice, where robust fluorescence remained visible up to 35 days following PCC1‐Cy5@PHCF‐Gel injection, while free PCC1‐Cy5 declined markedly by day 21 (Figure 4J,K). The strain‐dependent differences in signal persistence may be attributable to ocular pigmentation in C57BL/6J mice, which can partially mask fluorescence. Nevertheless, both models consistently demonstrated the superior intraocular retention and delivery efficiency of PCC1‐Cy5@PHCF‐Gel. Together with the LC‐QQQ‐MS data, these findings support sustained intraocular retention and ROS‐responsive release of PCC1 from PCC1/PHCF‐Gel, showing a markedly improved ocular residence profile compared with free PCC1 and thereby supporting its therapeutic potential for OFN.

### 2.4 PCC1/PHCF‐Gel‐Mediated Senolysis Alleviates Pathological Retinal Angiogenesis in the OIR Model

**FIGURE 5:**
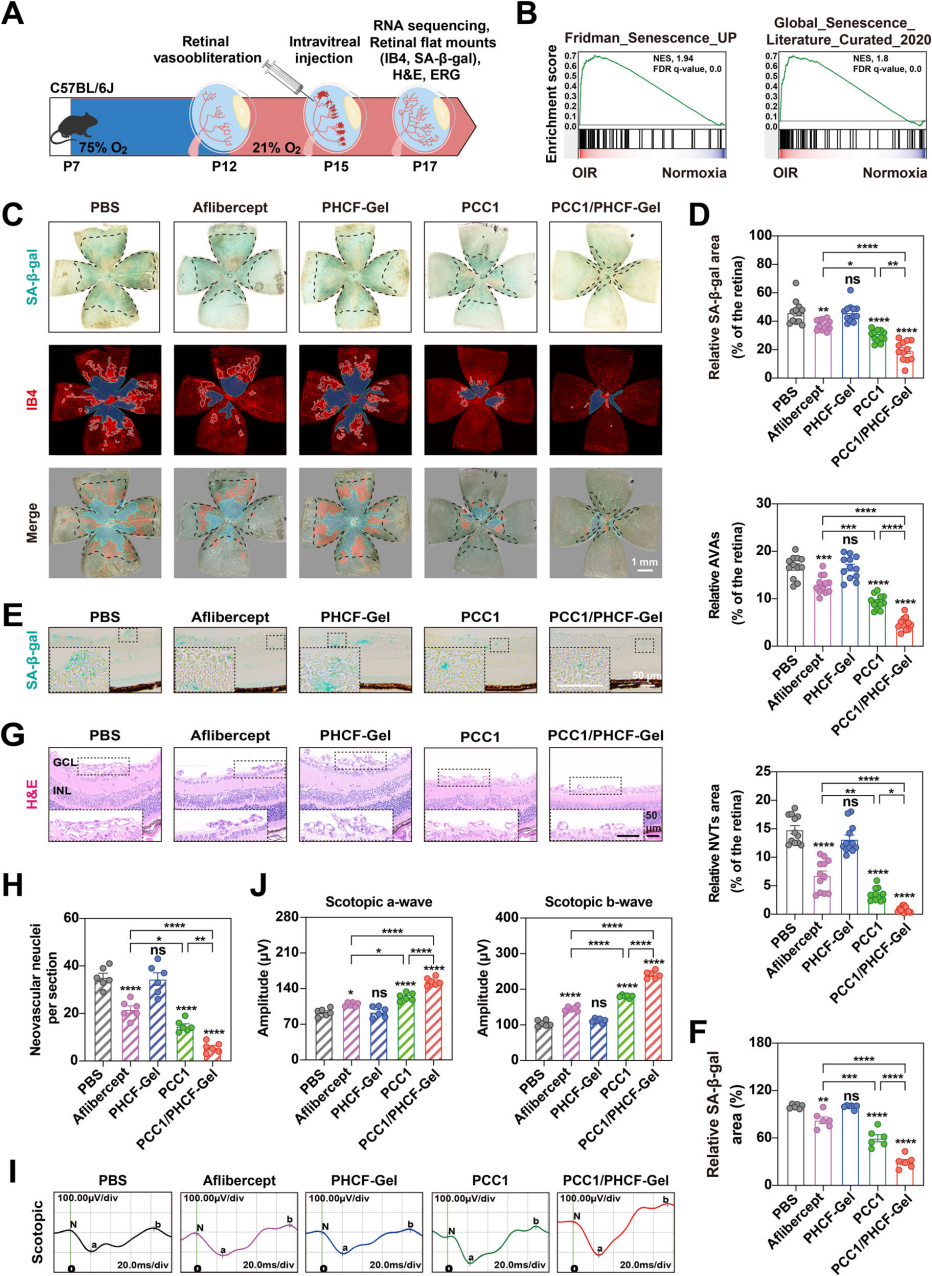
PCC1/PHCF‐Gel‐mediated senolysis alleviates pathological retinal angiogenesis in the OIR model. (A) Schematic of the experimental approach used to assess the therapeutic effects in the OIR model. (B) GSEA was performed on bulk retinal RNA‐seq data from normoxic (n = 2) and OIR (n = 3) mice to evaluate enrichment of senescence‐associated genes (e.g., Fridman_Senescence_UP and Global_Senescence_Literature_Curated_2020). NES, normalized enrichment score; FDR, false discovery rate. (C) SA‐β‐gal (blue‐green) and IB4 (red) staining of retinal flat mounts from P17 OIR mice 2 days after intravitreal injection with PBS, aflibercept, PHCF‐Gel, PCC1, and PCC1/PHCF‐Gel. In the IB4 images, AVAs and NVTs were pseudocolored in blue and red, respectively, and the overlap between SA‐β‐gal areas and IB4‐labeled vascular structures is shown. Scale bar: 1 mm. (D) Quantification of the AVAs, NVTs area, and SA‐β‐gal positive area in P17 OIR retinal flat mounts (*n* = 12). (E) Representative images of SA‐β‐Gal staining of retinal sagittal sections from P17 OIR mice 2 days after intravitreal injection with PBS, aflibercept, PHCF‐Gel, PCC1, and PCC1/PHCF‐Gel. Scale bar: 50 µm. (F) Quantitative analysis of the relative mean SA‐β‐gal staining area (*n* = 6). (G) Representative H&E‐stained micrographs of sagittal sections of NVTs in P17 OIR mouse retinas 2 days after intravitreal injection with PBS, aflibercept, PHCF‐Gel, PCC1, and PCC1/PHCF‐Gel. GCL, ganglion cell layer; INL, inner nuclear layer. Scale bar: 50 µm. (H) Quantification of preretinal neovascular nuclei per section in H&E‐stained sections (*n* = 6). (I) Representative scotopic ERG waves were obtained from P17 OIR mouse 2 days after intravitreal injection with PBS, aflibercept, PHCF‐Gel, PCC1, and PCC1/PHCF‐Gel. (J) Quantification of scotopic ERG waves (*n* = 6). All data are presented as means ± SEM. One‐way ANOVA followed by Tukey's post hoc test was used to determine the *p*‐values. ^*^ *p *< 0.05, ^**^ *p *< 0.01, ^***^ *p *< 0.001, and ^****^ *p* < 0.0001; ns, not significance.

An OIR mouse model mimicking the hallmarks of proliferative DR (PDR) and ROP, including ischemic avascular zones and pathological preretinal neovascularization, was then developed [30]. Briefly, neonatal mice were subjected to 75% hyperoxia from postnatal day 7 (P7)‐P12 to induce retinal vaso‐obliteration. Recovery under normoxic conditions (21% oxygen) triggered robust neovascularization, with peak vascular pathology observed on P17 [30]. Intravitreal intervention was administered at a predefined therapeutic window (P15) before the neovascularization peak (Figure 5A). To quantitatively analyze the retinal vascular morphology, we used isolectin B4 (IB4) for IF staining. Compared with normoxic controls, prominent retinal avascular areas (AVAs), representing ischemic–hypoxic regions, and characteristic neovascular tufts (NVTs) sprouting from the AVAs margins were observed at P17 in the OIR mice, confirming successful modeling (Figure S13A–C).

To investigate the association between retinal pathology and senescence in the OIR model, we performed bulk RNA‐seq of retinas from normoxic control and OIR mice at P17. Then, we conducted gene set enrichment analysis (GSEA) using cellular senescence‐associated gene sets (e.g., Fridman_Senescence_UP and Global_Senescence_Literature_Curated_2020) to interrogate the bulk RNA‐seq profiles, which revealed positive enrichment of senescence‐related signatures in OIR retinas compared with normoxic controls (Figure 5B) [15].

Previous studies showed that the peak of pathological neovascularization in OIR coincided with a maximal cellular senescence burden [19]. Consistently, SA‐β‐gal staining revealed senescence‐positive areas in both AVAs and NVTs regions in the retinas of OIR mice. This finding supported the concept that senescent cells are key components of pathological angiogenesis, consistent with previous reports (Figure S13A,B). Notably, ECs have been implicated as a major source of senescent cell in aging‐related vascular pathology [32]. Given the in vitro evidence that PCC1/PHCF‐Gel eliminated senescent HRMECs, we further assessed its in vivo therapeutic effects on retinal senescence. Compared with PBS controls, the senescence‐positive retinal area was reduced by 8.8%, 13.2%, and 23.6% in the aflibercept, PCC1, and PCC1/PHCF‐Gel groups, respectively. Unexpectedly, aflibercept also reduced the senescence‐associated retinal area, which differs from previous reports claiming no effect on retinal senescence [19]. This discrepancy may be attributed to its VEGF blockade, a key component of the SASP factors, thereby mitigating retinal senescence. In comparison, PCC1/PHCF‐Gel exhibited superior senolytic efficacy over PCC1 alone and aflibercept, highlighting its potential to remodel the senescence‐associated retinal microenvironment. Then, we also observed significant reductions in both the AVAs and NVTs in the aflibercept, PCC1, and PCC1/PHCF‐Gel‐treated groups compared with PBS controls on P17. Specifically, the total AVAs  was reduced by 3.7%, 7.4%, and 12.0%, respectively, in the aflibercept, PCC1, and PCC1/PHCF‐Gel groups; correspondingly, the number of NVTs decreased by 8.03%, 11.4%, and 14.0%, respectively. PHCF‐Gel alone exhibited no therapeutic effect. Importantly, PCC1/PHCF‐Gel demonstrated significantly greater efficacy than aflibercept or PCC1 monotherapy. PCC1 monotherapy also outperformed aflibercept, with all differences reaching statistical significance. Spatially, SA‐β‐gal–positive areas overlapped with IB4‐labeled vascular structures were predominantly distributed within NVTs and along AVAs margins. As senescence staining decreased, a proportional reduction in both NVTs and AVAs was observed (Figure 5C,D). This finding was further validated in retinal sagittal sections of OIR mice treated with PCC1/PHCF‐Gel, in which a marked reduction in blue‐stained senescent areas was accompanied by a proportional decrease in abnormal vascular structures (Figure 5E,F). These results confirmed that the neovascular regions and ischemic–hypoxic areas in the OIR retina were enriched with senescent cells. By clearing these senescent cells, PCC1/PHCF‐Gel significantly reduced pathological neovascularization and alleviated ischemic–hypoxia regions by promoting functional vessel ingrowth into the avascular zones, thereby providing a promising therapeutic strategy for OFN.

Furthermore, histopathological quantification of preretinal neovascular nuclei on H&E‐stained retinal sections revealed that PCC1/PHCF‐Gel treatment produced the greatest reduction in pathological neovascularization among the treatment groups, exceeding the effects of PCC1 monotherapy and aflibercept. This therapeutic superiority confirmed the significantly enhanced neovascular suppression efficacy of the sustained‐release gel formulation (Figure 5G,H).

To determine whether ameliorating pathological symptoms following PCC1/PHCF‐Gel treatment translated into improved retinal function, we performed whole‐field scotopic electroretinography (ERG) [33, 34]. Compared with retinas of normoxic mice, those of OIR mice exhibited reduced a‐wave and b‐wave amplitudes and had a significantly prolonged b‐wave implicit time (Figure S13D,E). The observed electrophysiological deviations were probably rooted in ischemia‐driven metabolic alterations of the photoreceptors, which hindered the outer segment disc membrane assembly and flattened the a‐wave. The diminished b‐wave amplitude was likely due to the injury sustained by bipolar neurons or the loosening of the synaptic bond between photoreceptors and their bipolar partners [29]. The prolongation of the b‐wave may reflect adaptive electrophysiological compensation mechanisms, such as reduced neurotransmitter recycling, that occur during ischemic stress [35]. These results suggested that retinal ischemia caused structural degeneration and dynamic neurophysiological remodeling. Notably, intravitreal administration of PCC1/PHCF‐Gel significantly enhanced both a‐wave and b‐wave amplitudes relative to the comparator groups, highlighting its potent neuroprotective effects on the retinal neural circuitry (Figure 5I,J). Taken together, PCC1/PHCF‐Gel alleviated vascular pathology by eliminating senescent cells, promoted reparative neovascularization in ischemic retinal tissue, and restored retinal function, illustrating its multi‐faceted therapeutic effects and clinical relevance in attenuating ischemic retinopathy.

### 2.5 Dual‐Target Senolysis of Senescent ECs and Microglia by PCC1/PHCF‐Gel via Apoptosis

**FIGURE 6:**
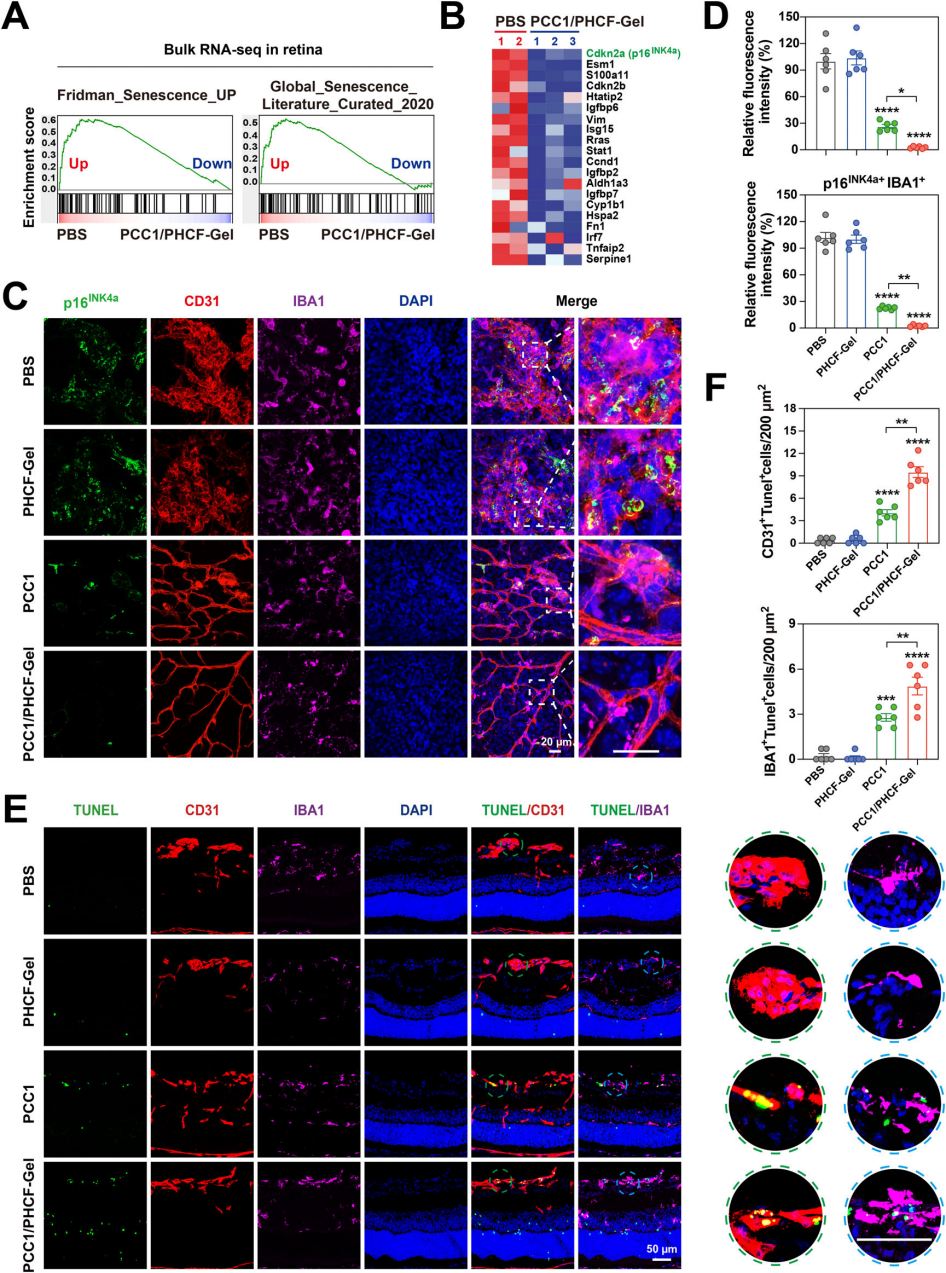
Dual‐target senolysis of senescent ECs and microglia by PCC1/PHCF‐Gel via apoptosis. (A) GSEA of bulk retinal RNA‐seq data comparing PBS‐treated OIR retinas (*n* = 2) with PCC1/PHCF‐Gel‐treated OIR retinas (*n* = 3) at P17. The analysis used senescence‐related gene sets, including Fridman_Senescence _UP and Global_Senescence_Literature_Curated_2020. (B) The heatmap highlights the top 20 DEGs from the Fridman_Senescence_UP gene set, based on bulk RNA‐seq data. (C) Confocal micrographs of retinal flat mounts showing robust staining of senescence markers p16^INK4a^ (green) colabeled with ECs (CD31; red), microglia (IBA1; purple), and nuclei (DAPI; blue). Representative images were taken from P17 OIR mouse retinas 2 days after intravitreal injection with PBS, aflibercept, PHCF‐Gel, PCC1, and PCC1/PHCF‐Gel. Scale bar, 20 µm. (D) Quantification is shown of p16^INK4a^^+^CD31^+^ and p16^INK4a^^+^IBA1^+^ relative fluorescence intensities from (C) (*n* = 6). (E) Confocal micrographs of the retinal sagittal sections showing TUNEL labeling (green) with CD31 (red), IBA1 (purple), and DAPI (blue) in P17 OIR mice 2 days after intravitreal injection with PBS, PCC1, PHCF‐Gel, and PCC1/PHCF‐Gel. Scale bar, 50 µm. (F) Quantification of TUNEL^+^CD31^+^ cells and TUNEL^+^IBA1^+^ cells per 200 µm² (*n* = 6). All data are presented as means ± SEM. One‐way ANOVA followed by Tukey's post hoc test was used to determine the *p*‐values. ^*^ *p* < 0.05, ^**^ *p* < 0.01, ^***^ *p* < 0.001, ^****^ *p* < 0.0001; ns, not significance.

To elucidate the mechanism by which PCC1/PHCF‐Gel alleviates pathological symptoms in ischemic retinopathy, we conducted GSEA on the ranked differentially expressed genes (DEGs) comparing PCC1/PHCF‐Gel‐treated retina with PBS control. In contrast to the PBS group, which positively correlated with cellular senescence‐related gene expression, the PCC1/PHCF‐Gel group showed a significant negative correlation with expression of senescence‐related genes in the Fridman_Senescence _UP and Global_Senescence_Literature_Curated_2020 gene sets (Figure 6A) and the SASP_Literature_Curated_UP, TNF, and NF‐κB signaling pathways [15, 16]. These findings suggest that PCC1/PHCF‐Gel may attenuate pathological retinal neovascularization by reducing senescent cell burden and suppressing SASP‐mediated TNF/NF‐κB inflammatory cytokine pathways (Figure S14A–C). Furthermore, a heatmap of the top 20 upregulated genes in PBS‐treated OIR retinas revealed prominent expression of classic senescence‐associated markers, including CDKN2A (encoding p16^INK4a^), which was markedly suppressed following PCC1/PHCF‐Gel treatment (Figure 6B and Figure S15).

In the OIR model, senescent ECs form the structural backbone of aberrant and leaky angiogenesis, while senescent microglia act as immune amplifiers that sustain the inflammatory‐angiogenic microenvironment. The spatial proximity of these two cell populations creates a vicious cycle that exacerbates pathological angiogenesis and hinders reparative vascular regeneration [36]. Following hyperoxia‐induced vaso‐obliterative subsequent return to normoxia, resting state microglia (characterized by highly ramified morphology) rapidly migrate toward ischemic regions and undergo morphological polarization (soma hypertrophy and process retraction) into an activated phenotype with localized aggregation [37]. Senescent microglia, which can exhibit an activated and dysfunctional state, have also been shown to drive retinopathy pathogenesis through IRE1α‐mediated unfolded protein response signaling [19]. Emerging evidence indicates that pharmacological depletion of microglia using targeted agents can suppresse pathological angiogenesis in experimental retinopathy models [38]. Collectively, the selective elimination of senescent ECs and microglia simultaneously may be a promising therapeutic strategy to suppress pathological angiogenesis while supporting reparative revascularization.

Subsequently, we performed IF staining using the senescence markers p16^INK4a^ and p21, the vascular marker CD31, the microglial marker IBA1, and DAPI (a nuclear marker), to identify alterations to senescent cells in vivo (Figure S16A,B). As shown in Figure 6C, representative confocal images of flat‐mounted OIR retinas at P17, obtained 2 days after intravitreal injection with PBS, aflibercept, PHCF‐Gel, PCC1, or PCC1/PHCF‐Gel, revealed marked accumulation of CD31^+^ cells co‐expressing p16^INK4a+^ or p21^+^ and IBA1^+^ cells co‐expressing p16^INK4a+^ or p21^+^ within NVTs in OIR retinas. However, these signals were markedly decreased in both the PCC1 and PCC1/PHCF‐Gel groups, with a more pronounced reduction in the PCC1/PHCF‐Gel group (Figure 6D and Figure S17A–C). A concomitant decrease in the spatial overlap between CD31 and IBA1 signals was observed in the PCC1 and PCC1/PHCF‐Gel groups and was accompanied by reduced NVTs complexity. Together, these findings suggest suppression of pathological neovascularization, consistent with our retinal vascular phenotyping results shown in Figure 4. Based on these results, PCC1/PHCF‐Gel not only inhibits pathological neovascularization but also reshapes the local microenvironment by eliminating senescent ECs and microglia. This dual clearance may attenuate inflammation and promote a more homeostatic retinal microenvironment, thereby supporting vascular repair, highlighting its potential as a superior therapeutic strategy.

We next sought to provide direct evidence that PCC1 induces senolysis through apoptosis. Previous studies have demonstrated that senescent ECs and microglia within NVTs in the OIR model exhibit an anti‐apoptotic state (Figure S18A). In our study, PCC1 treatment markedly increased apoptosis in these senescence‐enriched cell populations. This effect was validated by TUNEL staining combined with IF, which showed a significantly higher proportion of apoptotic ECs (CD31^+^TUNEL^+^) and microglia (IBA1^+^TUNEL^+^) after PCC1 and PCC1/PHCF‐Gel treatment. Moreover, PCC1/PHCF‐Gel induced more pronounced apoptosis than PCC1 alone, consistent with enhanced apoptotic clearance of ECs and microglia within senescence‐enriched lesions (Figure 6E,F). Collectively, these findings suggested that PCC1 suppresses pathological retinal neovascularization through apoptosis‐mediated senolysis, with the PCC1/PHCF‐Gel formulation exhibiting superior therapeutic effects.

### 2.6 scRNA‐seq Identifies CXCR4^+^ ECs as a Preferential Target of PCC1/PHCF‐Gel and Suggests Enhanced Revascularization

We conducted scRNA‐seq using the 10× Genomics platform to identify and characterize the endothelial and microglial subtypes altered by PCC1/PHCF‐Gel treatment. Retinas from OIR mice were harvested at P17 (2 days post‐injection) following intravitreal administration of PBS or PCC1/PHCF‐Gel. The retinas were processed as separate samples, and condition‐specific uniform manifold approximation and projection (UMAP) plots were generated following the Seurat workflow [39].

**FIGURE 7:**
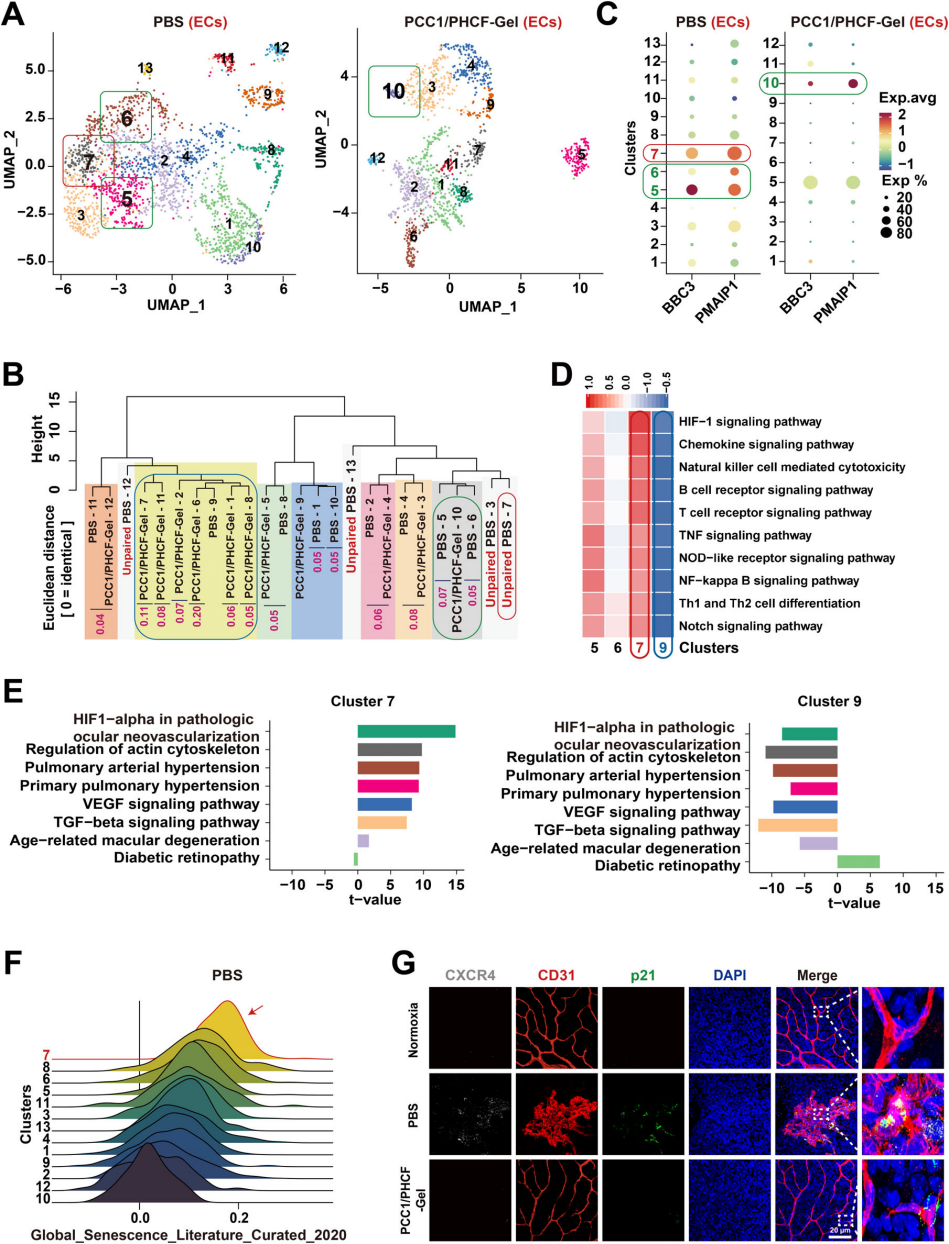
scRNA‐seq identifies CXCR4^+^ ECs as a preferential target of PCC1/PHCF‐Gel and suggests enhanced revascularization. (A) UMAP of scRNA‐seq analysis results comparing clusters of PBS‐treated (*n* = 3) and PCC1/PHCF‐Gel‐treated (*n* = 3) retinas on P17 of OIR modeling. (B) Hierarchical clustering dendrogram generated using ClusterMap v0.1.0 based on euclidean distances between cluster centroids. Similarity distances for matched pairs are shown in purple, and unpaired PBS clusters are labeled in red (0 = identical). (C) Dot plots showing expression of PMAIP1 (NOXA) and BBC3 (PUMA) across EC clusters in the PBS and PCC1/PHCF‐Gel groups (dot size, percentage of expressing cells; color, average expression). (D) GSVA heatmap for clusters 5, 6, 7, and 9 (clusters 7 and 9 highlighted). (E) Endothelial ‐specific pathway analysis comparing clusters 7 and 9 in the PBS‐treated group (t values shown). (F) Ridge plots visualizing the senescence score distributions across PBS‐treated endothelial clusters using Global_Senescence_Literature_Curated_2020 gene set. Red arrowhead denotes cluster 7. (G) Confocal micrographs of the retinal flat mounts showed the cluster 7 marker CXCR4 (gray) colabeled with ECs (CD31, red), senescence marker p21 (green), and nuclei (DAPI, blue) in normoxic controls and in OIR retinas harvested at P17, 2 days after intravitreal injection of PBS or PCC1/PHCF‐Gel. Scale bar, 20 µm.

To enable a comparison of endothelial heterogeneity, we integrated 3,832 ECs from the PBS and PCC1/PHCF‐Gel groups and analyzed them within a unified dimensionality‐reduction and clustering framework. UMAP visualization revealed a structured EC landscape in both conditions, with the PBS‐treated group resolved into 13 EC clusters, whereas the PCC1/PHCF‐Gel‐treated group resolving into 12 clusters, indicating a treatment‐associated shift in EC subpopulation composition (Figure 7A). Subsequently, all the clusters from both groups were combined. We then performed cross‐condition cluster matching using ClusterMap V0.1.0 software package by computing euclidean distances  to generate a hierarchical clustering map. Clusters between the groups with a ClusterMap: compare multiple scRNA‐seq datasets across different experimental conditions. Euclidean distance less than 0.25 were considered highly similar and were paired. When multiple clusters within the same group exhibited a euclidean distance less than 0.25, we first grouped them into a single category before performing inter‐group pairing. Based on the centroid‐distance criteria, several clusters within each condition were first merged into categories (e.g., PBS clusters 1 and 10; PBS clusters 5 and 6; and PCC1/PHCF‐Gel clusters 1, 2, 6, 7, 8, and 11) before cross‐condition pairing (Figure 7B). The specific cluster matching was as follows: the PBS category comprising clusters 1 and 10 was matched to PCC1/PHCF‐Gel cluster 9; the PBS category comprising clusters 5 and 6 was matched to PCC1/PHCF‐Gel cluster 10; and PBS cluster 9 was matched to the PCC1/PHCF‐Gel category comprising clusters 1, 2, 6, 7, 8, and 11. Ultimately, we identified seven matched cluster pairs between the PBS and PCC1/PHCF‐Gel groups. In addition, we identified four unique clusters (clusters 3, 7, 12, and 13) exclusively in the PBS group (Figure 7A,B).

Previous studies have shown that PCC1 selectively upregulates the proapoptotic proteins NOXA (PMAIP1) and PUMA (BBC3) in senescent cells [23]. Consistently, dot plot analysis revealed high expression of PMAIP1 and BBC3 in PBS‐treated clusters 5 and 6, as well as in the PBS‐specific (unpaired) cluster 7, and in the PCC1/PHCF‐Gel‐treated endothelial cluster 10 (Figure 7C). Notably, the numbers of cells in clusters 5 and 6, which corresponded to PCC1/PHCF‐Gel‐treated cluster 10, was markedly reduced (Figure 7A,B), suggesting that PCC1/PHCF‐Gel selectively eliminated most cells in these clusters. Furthermore, the unmatched cluster 7, which was present only in the PBS group, was no longer detected in the PCC1/PHCF‐Gel group (Figure 7C).

PCC1/PHCF‐Gel treatment enriched a major EC category comprising clusters 1, 2, 6, 7, 8, and 11, which mapped most closely to PBS cluster 9 based on centroid‐distance matching, suggesting a shift toward a more homeostatic, reparative‐like endothelial state. Gene set variation analysis (GSVA)‐based pathway analysis indicated that PBS clusters 5, 6, and 7 were positively associated with immune‐inflammatory pathways (e.g., cytokine signaling, pattern recognition receptor activation, and T‐cell differentiation), consistent with role in immune‐cell recruitment and inflammation regulation. In contrast, PBS cluster 9 exhibited significant negative associations with these pathways (Figure 7D). Moreover, endothelial‐specific analyses revealed that cluster 7 displayed the strongest enrichment of pathogenic pathways, particularly in HIF‐1α‐mediated pathological ocular neovascularization [15]. In contrast, cluster 9 showed a negative association relative to the other endothelial clusters (Figure 7E and Figure S19). Together, these data suggest that PCC1/PHCF‐Gel shifts EC composition away from proinflammatory/pathogenic programs and toward a cluster 9‐like transcriptional profile, which is consistent with our vascular phenotyping showing reduced avascular areas in OIR retinas (Figure 5C,D). Accordingly, PCC1/PHCF‐Gel may facilitate revascularization within ischemic‐hypoxic regions, thereby alleviating ischemia‐driven pathological angiogenesis.

The senescence scoring analysis based on genes from the Fridman_Senescence_UP and Global_Senescence_Literature_Curated_2020 gene sets revealed that senescence‐associated signatures were predominantly enriched in PBS‐treated clusters 5 and 6, as well as the PBS‐specific cluster 7 [40]. Among these, PBS‐specific cluster 7 (167 cells; 7% of ECs in the PBS group) displayed the strongest senescence characteristics (Figure 7F and Figure S20). By contrast, cluster 9 in the PCC1/PHCF‐Gel‐treated group exhibited a markedly reduced senescence score, indicating effective suppression of senescence signatures following treatment.

We identified the top 10 markers specific to cluster 7 (Table S2). Confocal IF images showed that the ECs top‐ranked markers, CXCR4 and p21 (a senescence marker), were colocalized in pathological neovascular lesions but were almost undetectable in the PCC1/PHCF‐Gel‐treated groups (Figure 7G). Notably, CXCR4 and p21 signals were undetectable in normoxic retinas, indicating that PCC1/PHCF‐Gel selectively targets a lesion‐associated CXCR4^+^p21^+^ senescent EC subpopulation that is absent or minimal in healthy retinal vasculature.

### 2.7 scRNA‐seq Reveals Selective Elimination of IFITM3^+^ Microglia

**FIGURE 8:**
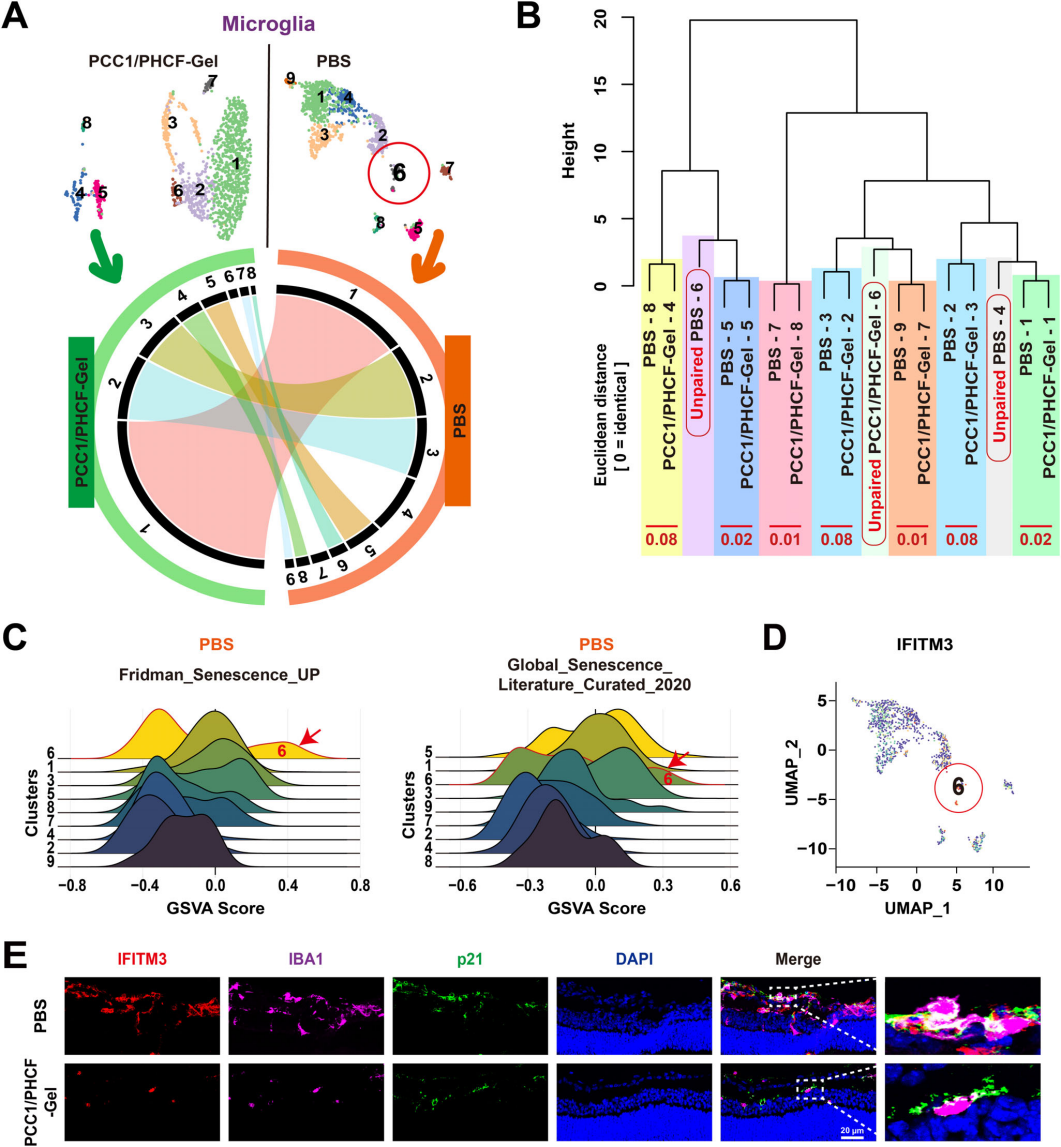
scRNA‐seq reveals selective elimination of IFITM3^+^ microglia. (A) UMAP plots (top) of microglia from P17 OIR retinas harvested 2 days after intravitreal injection of PBS or PCC1/PHCF‐Gel, and a Circos plot (bottom) illustrating cross‐condition cluster linkages; chords connect clusters with similar transcriptional/marker‐gene signatures. (B) Euclidean centroid distance‐based hierarchical clustering dendrogram of microglial clusters from PBS and PCC1/PHCF‐Gel groups (0 = identical). Centroid distances for matched cluster pairs are shown in red, and unpaired clusters are indicated. (C) Ridge plots illustrating the distribution of GSVA senescence scores across retinal clusters for PBS group microglia, calculated using the Fridman_Senescence_UP and Global_Senescence_Literature_Curated_2020 signatures gene sets. The arrow denotes cluster 6. (D) Feature plot showing IFITM3 expression across microglial UMAP clusters; cluster 6 is highlighted. (E) Confocal micrographs of the retinal sagittal sections displayed the cluster 6 marker IFITM3 (red) colabeled with microglia (IBA1, purple), p21 (green), and nuclei (DAPI, blue) in P17 OIR mice 2 days after intravitreal administration of PBS and PCC1/PHCF‐Gel. Scale bar, 20 µm.

To further clarify the microglial cell populations, we analyzed 2,523 microglia from PBS‐ and PCC1/PHCF‐Gel‐treated retinas with Circos linkages and euclidean‐based hierarchical trees (Figure 8A,B) [41, 42]. The analysis pinpointed seven paired clusters; among them, clusters 4 and 6 were unique to the PBS group (Figure 8B). We calculated senescence scores across all clusters using the Fridman_Senescence_UP and Global_Senescence_Literature_Curated_2020 gene sets. PBS‐specific cluster 6 exhibited a robust senescence‐associated signature (Figure 8C), and differential expression analysis identified IFITM3 as its representative marker (Figure 8D). Mechanistically, IFITM3 has been reported to couple innate immune activation to downstream pathological processes by directly modulating γ‐secretase activity, thereby linking inflammatory signaling to proteolytic/amyloidogenic pathways and sustained tissue stress [43]. Consistent with this framework, IFITM3 is induced in aged brains after ischemic stroke and is robustly upregulated in primary microglia exposed to inflammatory stimuli, supporting its role as a stress‐responsive effector in activated microglia [44]. Together, these observations support a model in which ROS‐rich chronic stress engages innate immune sensing and type I interferon programs, driving an IFITM3‐high, inflammation‐prone microglial state that can overlap with senescence‐associated transcriptional features. Accordingly, IFITM3 likely contributes to the functional output of “senescence‐associated” microglia by amplifying IFN/inflammatory signaling and downstream stress pathways, but should be interpreted alongside senescence gene‐set enrichment and canonical senescence markers rather than as a standalone indicator of microglial senescence. Consistent with this interpretation, confocal IF of IFITM3 and p21 showed colocalization within pathological neovascular lesions in PBS‐treated retinas, and both signals were markedly reduced after PCC1/PHCF‐Gel treatment (Figure 8E).

The data demonstrated that PCC1/PHCF‐Gel selectively eliminated pathological senescent ECs and microglial subclusters, consistent with attenuation of aberrant neovascularization. By removing these senescent populations, PCC1/PHCF‐Gel alleviates pathological retinal angiogenesis through a dual anti‐senescence mechanism that not only suppresses aberrant vessel formation but also resolves the pro‐inflammatory microenvironment sustained by senescent microglia. In parallel, PCC1/PHCF‐Gel shifted EC composition toward a more reparative‐like state, supporting vascular remodeling in ischemic retina. Collectively, these findings highlight PCC1/PHCF‐Gel as a dual‐target senolytic strategy with translational potential for ocular neovascular diseases.

### 2.8 PCC1/PHCF‐Gel Mediates Senolysis to Inhibit Laser‐Induced CNV

**FIGURE 9:**
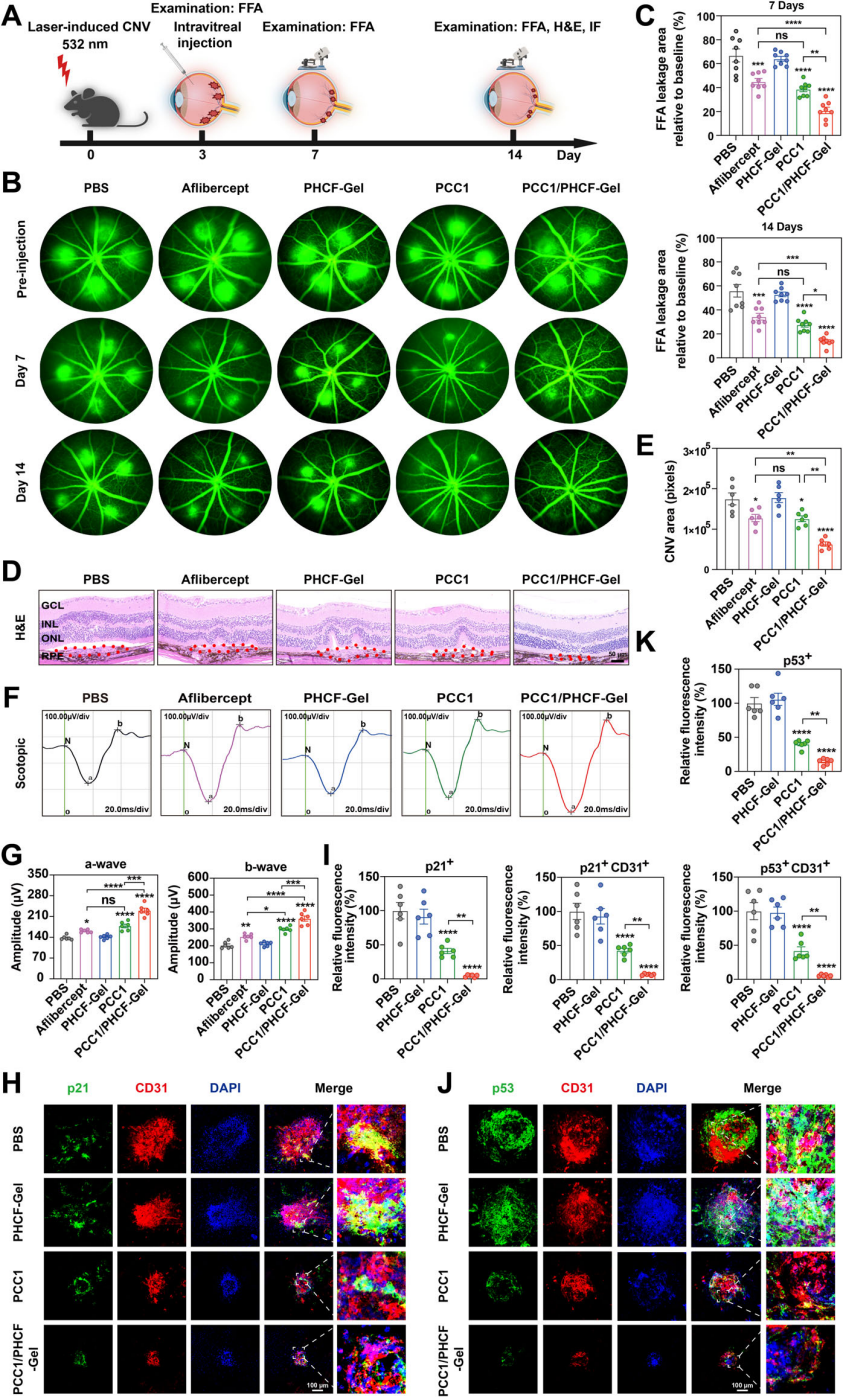
PCC1/PHCF‐Gel mediates senolysis to inhibit laser‐induced CNV. (A) Schematic of the laser‐induced CNV model and experimental design. (B) Representative FFA images of the laser‐induced CNV mouse model were captured at 7 and 14 days after intravitreal injection of PBS, aflibercept, PHCF‐Gel, PCC1, or PCC1/PHCF‐Gel administered on day 3. (C) Quantification of FFA leakage area normalized to the baseline (*n* = 8). (D) Representative H&E stained images of transverse sections of the CNV model retinas were captured 14 days after intravitreal injection of PBS, aflibercept, PHCF‐Gel, PCC1, or PCC1/PHCF‐Gel. Scale bar, 50 µm. (E) Quantification of CNV area (pixels) in the H&E‐stained images (*n* = 6). (F) Representative scotopic ERG traces recorded at day 14 after the intravitreal injection of PBS, Aflibercept, PHCF‐Gel, PCC1, or PCC1/PHCF‐Gel (*n* = 6). (G) Quantification of scotopic a‐wave and b‐wave amplitudes at day 14 (*n* = 6). (H,J) Confocal micrographs of choroidal flat mounts at day 14 post‐laser induction of CNV, showing IF for the senescence markers p21 (H, green) and p53 (J, green), the neovascular marker CD31 (red), and nuclei (DAPI, blue). Mice received intravitreal injection of PBS, PHCF‐Gel, PCC1, or PCC1/PHCF‐Gel. Scale bars, 100um. (I,K) Relative fluorescence intensities of p21 (H) and p53 (J) were analyzed (*n* = 6). All data are presented as means ± SEM. One‐way ANOVA followed by Tukey's post hoc test was used to determine the *p*‐values. ^*^ *p* < 0.05, ^**^ *p *< 0.01, ^***^ *p* < 0.001, ^****^ *p* < 0.0001; ns, not significance.

The anti‐senescence activity of PCC1/PHCF‐Gel was supported by findings in the OIR model, where treatment not only suppressed pathological angiogenesis but also provided neuroprotection by remodeling the diseased microenvironment. Building on these findings, we next investigated whether PCC1/PHCF‐Gel could attenuate senescence in CNV lesions. To this end, a two‐week in vivo study was conducted in a laser‐induced CNV mouse model to assess the therapeutic efficacy of intravitreal PCC1/PHCF‐Gel for nAMD (Figure 9A). CNV was induced using standard 532‐nm laser photocoagulation, and successful model establishment was confirmed at day 3 by fundus fluorescein angiography (FFA), fundus photography, optical coherence tomography, and H&E staining [45]. FFA revealed pronounced vascular leakage, indicative of increased vascular permeability (Figure S21A), while all imaging and histological modalities consistently demonstrated progressive expansion of CNV lesions in model mice (Figure S21B,C).

To further investigate the therapeutic efficacy of PCC1/PHCF‐Gel in the CNV model, FFA was employed to quantify CNV‐associated fluorescein leakage. In nAMD, pathological neovascularization is characterized by the formation of new vessels with increased microvascular permeability, often leading to retinal hemorrhage. At day 3 (pre‐injection baseline), lesion areas was comparable across all five groups, confirming successful model induction and a well‐matched baseline. By day 7, FFA revealed significantly reduced CNV leakage in the aflibercept, PCC1, and PCC1/PHCF‐Gel groups compared with PBS. Notably, PCC1/PHCF‐Gel produced a greater reduction in leakage than either PCC1 or aflibercept, although PCC1 alone was as effective as aflibercept. The superior therapeutic response of PCC1/PHCF‐Gel persisted through day 14. In contrast, PHCF‐Gel alone failed to produce therapeutic benefit at either day 7 or day 14 (Figure 9B,C). Collectively, these findings demonstrated that PCC1/PHCF‐Gel more effectively inhibited neovascularization and prevented vascular leakage than first‐line anti‐VEGF therapy or PCC1 alone.

Consistent with the FFA findings, fundus photography revealed that laser‐induced CNV lesions appeared as circular regions noticeably paler than the surrounding normal retina (Figure S21B). The total cross‐sectional area of CNV was smaller in the PCC1/PHCF‐Gel group than in both the PCC1 and aflibercept groups, and was the smallest among all five treatment groups, as demonstrated by H&E analyse (Figure 9D,E). These results indicated that PCC1/PHCF‐Gel exerted a more robust therapeutic effect than either aflibercept or PCC1 alone, even after 14 days of treatment. No significant differences were observed between the PCC1 and aflibercept groups. These imaging and histological results underscore the superior efficacy of PCC1/PHCF‐Gel in suppressing CNV progression.

Clinically, nAMD is characterized by progressive macular damage and central vision loss caused by abnormal neovascularization that extends into the retina. This new vessel results in fluid or blood leakage and scarring, leading to rapid central vision loss and photoreceptor degeneration [4]. To evaluate the extent of preserved photoreceptor function, ERG was performed. Recordings from mice without laser induction, which did not develop neovascularization, were used as the normal retinal baseline. Three days after laser‐induced CNV, ERG analyses revealed marked impairment of scotopic response in model mice, confirming compromised retinal function (Figure S21D,E). ERG was performed at day 14 after laser induction to assess the therapeutic effects of various treatments. Consistent with the lesion imaging and histological findings, mice treated with PCC1/PHCF‐Gel exhibited significantly stronger scotopic ERG response than the other groups, with increased a‐ and b‐wave amplitudes PCC1/PHCF‐Gel consistently alleviated CNV‐associated reductions in ERG amplitudes throughout the two‐week treatment period and achieved greater functional recovery than PCC1 alone (Figure 9F,G).

To further explore the therapeutic mechanism, multiplex IF staining was performed, which revealed senescence markers (p21 and p53) co‐localized with vascular ECs (CD31) in CNV lesions (Figure 9H,J). The results showed a significant accumulation of senescent ECs (CD31^+^p21^+^ or CD31^+^p53^+^) within the neovascular networks of CNV lesions. Treatment with PCC1/PHCF‐Gel markedly reduced the number of senescent ECs and significantly outperformed PCC1 monotherapy. In contrast, no significant difference in senescent cell numbers was observed between the PHCF‐Gel and PBS groups, confirming the biological inertness of the hydrogel carrier (Figure 9I,K).

These findings indicate that PCC1/PHCF‐Gel effectively promotes retinal repair. Collectively, our results demonstrate that PCC1/PHCF‐Gel attenuates CNV progression and reduces vascular leakage by targeting and eliminating senescent ECs, thereby providing structural and functional rescue in laser‐induced CNV models.

## 3 Conclusions

Therapies targeting pathological angiogenesis have shown substantial efficacy in ocular neovascular diseases such as OFN. However, these approaches generally fail to distinguish between physiological and diseased vasculature, leading to off‐target adverse effects. In this study, we demonstrate that pathological vessels in OFN are characterized by senescent cells within the vascular unit and can be eliminated through therapeutic senolysis.

To advance a translatable senescence‐targeted therapy for OFN, we engineered a ROS‐responsive hydrogel system (PCC1/PHCF‐Gel) that enabled long‐term, sustained, and lesion‐localized release of the senolytic agent PCC1. PCC1 exerted a dual‐target anti‐senescence mechanism by selectively eliminating senescent ECs and microglia via apoptosis, thereby disrupting the local pathological vascular–inflammatory feedback loop that drives neovascular progression. Single‐cell sequencing combined with transcriptomic analysis further revealed that PCC1/PHCF‐Gel cleared specific senescent subpopulations, including CXCR4^+^ senescent ECs and IFITM3^+^ senescent microglia, both absent in healthy retinal tissue. This targeted senolysis remodeled the diseased microenvironment, facilitated vascular unit reorganization, and ultimately enabled the regeneration of functional blood vessels in the avascular and ischemic retina.

The ocular fundus vasculature can be remodeled and restored through targeted senolysis, as senescent cells within pathological blood vessels drive disease progression. Unlike single‐factor inhibition (e.g., anti‐VEGF therapies), eliminating these senescent cells holistically addresses the source of pathogenic SASP factors by halting further vascular degeneration and promoting tissue microenvironment rejuvenation, thus enabling structural and functional disease modification. The data we presented showed that PCC1/PHCF‐Gel holds promise as a therapeutic option for managing senescent cell‐dependent ocular fundus conditions, particularly PDR and nAMD. The formulation we propose is relatively simple to scale up, which is advantageous for broader application. Furthermore, the reagents used in our PHCF‐Gel system have been granted clinical use approvals and have minimal safety concerns, suggesting that PCC1/PHCF‐Gel has great potential for use in clinical settings as an innovative treatment for pathological OFN.

## Conflicts of Interest

The authors declare no conflicts of interest.

## Supporting information




**Supporting File 1**: advs73722‐sup‐0001‐SuppMat.docx.

**Supporting File 2**: advs73722‐sup‐0002‐VideoS1.mp4.

**Supporting File 3**: advs73722‐sup‐0003‐VideoS2.mp4.

## Data Availability

The data that support the findings of this study are available from the corresponding author upon reasonable request.

## References

[1] G. A. Lutty and D. S. McLeod , “Development of the Hyaloid, Choroidal and Retinal Vasculatures in the Fetal human Eye,” Progress in Retinal and Eye Research 62 (2018): 58–76, 10.1016/j.preteyeres.2017.10.001. 29081352 PMC5776052

[2] S. X. Zhang and J. Ma , “Ocular Neovascularization: Implication of Endogenous Angiogenic Inhibitors and Potential Therapy,” Progress in Retinal and Eye Research 26 (2007): 1–37, 10.1016/j.preteyeres.2006.09.002. 17074526

[3] M. Fevereiro‐Martins , C. Marques‐Neves , H. Guimarães , and M. Bicho , “Retinopathy of Prematurity: a Review of Pathophysiology and Signaling Pathways,” Survey of Ophthalmology 68 (2023): 175–210, 10.1016/j.survophthal.2022.11.007. 36427559

[4] R. H. Guymer and T. G. Campbell , “Age‐related Macular Degeneration,” The Lancet 401 (2023): 1459–1472, 10.1016/S0140-6736(22)02609-5. 36996856

[5] J. W. Y. Yau , S. L. Rogers , R. Kawasaki , et al., “Global Prevalence and Major Risk Factors of Diabetic Retinopathy,” Diabetes Care 35 (2012): 556–564, 10.2337/dc11-1909. 22301125 PMC3322721

[6] K. Sabri , A. L. Ells , E. Y. Lee , S. Dutta , and A. Vinekar , “Retinopathy of Prematurity: a Global Perspective and Recent Developments,” Pediatrics 150 (2022): 2021053924. 10.1542/peds.2021-053924 35948728

[7] M. J. Elman , “All Patients Treated for Proliferative Diabetic Retinopathy Need to Be Monitored Carefully over Time for Further Treatment,” Ophthalmology 128 (2021): 1458–1459, 10.1016/j.ophtha.2021.07.019. 34556311

[8] W. L. Wong , X. Su , X. Li , et al., “Global Prevalence of Age‐related Macular Degeneration and Disease Burden Projection for 2020 and 2040: a Systematic Review and Meta‐analysis,” The Lancet Global Health 2 (2014): e106–e116, 10.1016/S2214-109X(13)70145-1. 25104651

[9] R. N. Israilevich , H. Mansour , S. N. Patel , et al., “Risk of Endophthalmitis Based on Cumulative Number of Anti‐VEGF Intravitreal Injections,” Ophthalmology 131 (2024): 667–673, 10.1016/j.ophtha.2023.12.033. 38182029

[10] A. Shaheen , D. Mehra , S. Ghalibafan , et al., “Efficacy and Safety of Anti‐VEGF Injections and Surgery for Age‐Related Macular Degeneration‐Related Submacular Hemorrhage,” Ophthalmology Retina 9 (2025): 4–12, 10.1016/j.oret.2024.07.024. 39098637

[11] A. Uemura , M. Fruttiger , P. A. D'Amore , et al., “VEGFR1 signaling in Retinal Angiogenesis and Microinflammation,” Progress in Retinal and Eye Research 84 (2021): 100954, 10.1016/j.preteyeres.2021.100954. 33640465 PMC8385046

[12] A. F. Alsoudi , K. M. Wai , E. Koo , R. Parikh , P. Mruthyunjaya , and E. Rahimy , “Initial Therapy of Panretinal Photocoagulation vs Anti‐VEGF Injection for Proliferative Diabetic Retinopathy,” JAMA Ophthalmology 142 (2024): 972–975, 10.1001/jamaophthalmol.2024.3283. 39207799 PMC11362971

[13] R. N. Khurana , C. Li , and F. Lum , “Loss to Follow‐up in Patients with Neovascular Age‐Related Macular Degeneration Treated with Anti–VEGF Therapy in the United States in the IRIS Registry,” Ophthalmology 130 (2023): 672–683, 10.1016/j.ophtha.2023.02.021. 36858288

[14] M.‐M. Macaron , N. Al Sabbakh , M. Z. Shami , et al., “Anti‐VEGF Injections vs. Panretinal Photocoagulation Laser Therapy for Proliferative Diabetic Retinopathy,” Ophthalmology Retina 9 (2025): 105–121, 10.1016/j.oret.2024.08.004. 39128789

[15] S. Crespo‐Garcia , P. R. Tsuruda , A. Dejda , et al., “Pathological Angiogenesis in Retinopathy Engages Cellular Senescence and Is Amenable to Therapeutic Elimination via BCL‐xL Inhibition,” Cell Metabolism 33 (2021): 818–832.e7, 10.1016/j.cmet.2021.01.011. 33548171

[16] F. Binet , G. Cagnone , S. Crespo‐Garcia , et al., “Neutrophil Extracellular Traps Target Senescent Vasculature for Tissue Remodeling in Retinopathy,” Science 369 (2020): aay5356. 10.1126/science.aay5356 32820093

[17] B. Wang , J. Han , J. H. Elisseeff , and M. Demaria , “The Senescence‐associated Secretory Phenotype and Its Physiological and Pathological Implications,” Nature Reviews Molecular Cell Biology 25 (2024): 958–978, 10.1038/s41580-024-00727-x. 38654098

[18] E. G. O'Koren , C. Yu , M. Klingeborn , et al., “Microglial Function Is Distinct in Different Anatomical Locations during Retinal Homeostasis and Degeneration,” Immunity 50 (2019): 723–737. 30850344 10.1016/j.immuni.2019.02.007 PMC6592635

[19] M. Oubaha , K. Miloudi , A. Dejda , et al., “Senescence‐associated Secretory Phenotype Contributes to Pathological Angiogenesis in Retinopathy,” Science Translational Medicine 8 (2016): 362ra144, 10.1126/scitranslmed.aaf9440. 27797960

[20] M. Hata , M. Hata , E. M. M. A. Andriessen , et al., “Early‐Life Peripheral Infections Reprogram Retinal Microglia and Aggravate Neovascular Age‐Related Macular Degeneration in Later Life,” Journal of Clinical Investigation 133 (2023): 159757. 10.1172/JCI159757 PMC9927938 36787231

[21] K. Cui , X. Tang , A. Hu , et al., “Therapeutic Benefit of Melatonin in Choroidal Neovascularization during Aging through the Regulation of Senescent Macrophage/Microglia Polarization,” Investigative Opthalmology & Visual Science 64 (2023): 19, 10.1167/iovs.64.11.19. PMC10431207 37578424

[22] S. Crespo‐Garcia , F. Fournier , R. Diaz‐Marin , et al., “Therapeutic Targeting of Cellular Senescence in Diabetic Macular Edema: Preclinical and Phase 1 Trial Results,” Nature Medicine 30 (2024): 443–454, 10.1038/s41591-024-02802-4. 38321220

[23] Q. Xu , Q. Fu , Z. Li , et al., “The Flavonoid Procyanidin C1 Has Senotherapeutic Activity and Increases Lifespan in Mice,” Nature Metabolism 3 (2021): 1706–1726, 10.1038/s42255-021-00491-8. PMC8688144 34873338

[24] Y. Liu , X. Liu , X. Chen , et al., “Senolytic and Senomorphic Agent Procyanidin C1 Alleviates Structural and Functional Decline in the Aged Retina,” Proceedings of the National Academy of Sciences 121 (2024): 2311028121, 10.1073/pnas.2311028121. PMC11067450 38657052

[25] Y. Gan , K. Wang , X. Chen , et al., “Senolytic Procyanidin C1 Alleviates Renal Fibrosis by Promoting Apoptosis of Senescent Renal Tubular Epithelial Cells,” The FASEB Journal 39 (2025): 70362, 10.1096/fj.202402558R. 39878685

[26] M. Shao , Y. Qiu , M. Shen , et al., “Procyanidin C1 Inhibits Bleomycin‐Induced Pulmonary Fibrosis in Mice by Selective Clearance of Senescent Myofibroblasts,” The FASEB Journal 38 (2024): 23749, 10.1096/fj.202302547RR.38953707

[27] Y. Zhang , X. Zhou , G. Liang , et al., “Iron‐Chelating and ROS‐Scavenging Polymers with Thioketal and Thioether Bonds Delivering Ferroptosis Inhibitor Lip‐1 Provide a Triple Therapeutic Strategy for Retina Ganglion Cells in Acute Glaucoma,” Advanced Materials 2507526. 10.1002/adma.202507526 40641252

[28] D. Zhao , J. Zhang , C. Chen , et al., “Rejuvenation Modulation of Nucleus Pulposus Progenitor Cells Reverses Senescence‐Associated Intervertebral Disc Degeneration,” Advanced Materials 37 (2025): 2409979, 10.1002/adma.202409979. 39969420

[29] H. Gao , M. Chen , Y. Liu , et al., “Injectable Anti‐Inflammatory Supramolecular Nanofiber Hydrogel to Promote Anti‐VEGF Therapy in Age‐Related Macular Degeneration Treatment,” Advanced Materials 35 (2023): 2204994, 10.1002/adma.202204994. 36349821

[30] K. M. Connor , N. M. Krah , R. J. Dennison , et al., “Quantification of Oxygen‐induced Retinopathy in the Mouse: a Model of Vessel Loss, Vessel Regrowth and Pathological Angiogenesis,” Nature Protocols 4 (2009): 1565–1573, 10.1038/nprot.2009.187. 19816419 PMC3731997

[31] X. Li , H. Zeng , L. Zhang , J. Zhang , Y. Guo , and J. Leng , “An Integrated LC‐MS/MS Platform for Noninvasive Urinary Nucleus Acid Adductomics: a Pilot Study for Tobacco Exposure,” Journal of Hazardous Materials 474 (2024): 134780, 10.1016/j.jhazmat.2024.134780. 38861899

[32] S. Y. Kim and J. Cheon , “Senescence‐associated Microvascular Endothelial Dysfunction: a Focus on the Blood‐brain and Blood‐retinal Barriers,” Ageing Research Reviews 100 (2024): 102446, 10.1016/j.arr.2024.102446. 39111407

[33] S. Li , D. Sun , S. Chen , et al., “UCP2–SIRT3 Signaling Relieved Hyperglycemia‐Induced Oxidative Stress and Senescence in Diabetic Retinopathy,” Investigative Opthalmology & Visual Science 65 (2024): 14, 10.1167/iovs.65.1.14. PMC10774691 38175638

[34] S. Chen , D. Sun , S. Zhang , et al., “TIN2 modulates FOXO1 Mitochondrial Shuttling to Enhance Oxidative Stress‐induced Apoptosis in Retinal Pigment Epithelium under Hyperglycemia,” Cell Death & Differentiation 31 (2024): 1487–1505, 10.1038/s41418-024-01349-8. 39080375 PMC11519896

[35] Y. Liu , C. M. McDowell , Z. Zhang , H. E. Tebow , R. J. Wordinger , and A. F. Clark , “Monitoring Retinal Morphologic and Functional Changes in Mice Following Optic Nerve Crush,” Investigative Opthalmology & Visual Science 55 (2014): 3766–3774, 10.1167/iovs.14-13895. 24854856

[36] Q. Bai , X. Wang , H. Yan , et al., “Microglia‐Derived Spp1 Promotes Pathological Retinal Neovascularization via Activating Endothelial Kit/Akt/mTOR Signaling,” Journal of Personalized Medicine 13 (2023): 146, 10.3390/jpm13010146. 36675807 PMC9866717

[37] S. Ben , Y. Ma , Y. Bai , et al., “Microglia‐endothelial Cross‐talk Regulates Diabetes‐induced Retinal Vascular Dysfunction through Remodeling Inflammatory Microenvironment,” Iscience 27 (2024): 109145, 10.1016/j.isci.2024.109145. 38414848 PMC10897849

[38] K. A. Church , D. Rodriguez , D. Vanegas , et al., “Models of Microglia Depletion and Replenishment Elicit Protective Effects to Alleviate Vascular and Neuronal Damage in the Diabetic Murine Retina,” Journal of Neuroinflammation 19 (2022): 300, 10.1186/s12974-022-02659-9. 36517889 PMC9753268

[39] T. Stuart , A. Butler , P. Hoffman , et al., “Comprehensive Integration of Single‐Cell Data,” Cell 177 (2019): 1888–1902.e21, 10.1016/j.cell.2019.05.031. 31178118 PMC6687398

[40] C. Hafemeister and R. Satija , “Normalization and Variance Stabilization of Single‐cell RNA‐seq Data Using Regularized Negative Binomial Regression,” Genome Biology 20 (2019): 296, 10.1186/s13059-019-1874-1. 31870423 PMC6927181

[41] I. Tirosh , B. Izar , S. M. Prakadan , et al., “Dissecting the Multicellular Ecosystem of Metastatic Melanoma by Single‐cell RNA‐seq,” Science 352 (2016): 189–196, 10.1126/science.aad0501. 27124452 PMC4944528

[42] X. Gao , D. Hu , M. Gogol , and H. Li , “ClusterMap: compare multiple single cell RNA‐Seq datasets across different experimental conditions,” Bioinformatics 17(2019): 3038–3045. 10.1093/bioinformatics/btz024 30649203

[43] J.‐Y. Hur , G. R. Frost , X. Wu , et al., “The Innate Immunity Protein IFITM3 Modulates γ‐secretase in Alzheimer's Disease,” Nature 586 (2020): 735–740, 10.1038/s41586-020-2681-2. 32879487 PMC7919141

[44] E. Harmon , A. Doan , J. Bautista‐Garrido , J. E. Jung , S. P. Marrelli , and G. S. Kim , “Increased Expression of Interferon‐Induced Transmembrane 3 (IFITM3) in Stroke and Other Inflammatory Conditions in the Brain,” International Journal of Molecular Sciences 23 (2022): 8885, 10.3390/ijms23168885. 36012150 PMC9408431

[45] V. Lambert , J. Lecomte , S. Hansen , et al., “Laser‐induced Choroidal Neovascularization Model to Study Age‐related Macular Degeneration in Mice,” Nature Protocols 8 (2013): 2197–2211, 10.1038/nprot.2013.135. 24136346

