# Supplementary material for: Dual‐Target ROS‐Driven Spatiotemporal Senolysis for Vascular Repair and Immune Microenvironment Reprogramming in the Treatment of Ocular Fundus Neovascularization
Source: Adv Sci (Weinh). 2026 Feb 16;13(16):e23495. doi: 10.1002/advs.202523495 (PMC13042622; doi:10.1002/advs.202523495)
Supplement: Supplementary file 1 — Supporting File 1: advs73722‐sup‐0001‐SuppMat.docx. [file ADVS-13-e23495-s002.docx]

Supporting Information

**Dual-Target ROS-Driven Spatiotemporal Senolysis for Vascular Repair and Immune Microenvironment Reprogramming in the Treatment of Ocular Fundus Neovascularization**

*Yali Zhou, Tianxing Chen, Peiyu Liu, Kangjia Lv, Yifan Wang, Xiaoqian Wang, Junwei Fang, Chong Chen,* Zhaoyang Wang,* Fang Wei,* and Xun Xu**

1. Zhou, P. Liu, K. Lv, Y. Wang, X. Wang, J. Fang, C. Chen, F. Wei, X. Xu

Department of Ophthalmology, Shanghai General Hospital, Shanghai JiaoTong University School of Medicine

National Clinical Research Center for Eye Diseases

Shanghai Clinical Research Center for Eye Diseases

Shanghai Key Clinical Specialty, Shanghai Key Laboratory of Ocular Fundus Diseases

Shanghai Engineering Center for Visual Science and Photomedicine

Shanghai Engineering Center for Precise Diagnosis and Treatment of Eye Diseases

Shanghai 200080, China

E-mail: [chong.chen1@shgh.cn;](mailto:chong.chen1@shgh.en;) [weifang73@hotmail.com](mailto:weifang73@126.com); [drxuxun@sjtu.edu.cn](mailto:drxuxun@sjtu.edu.cn)

T. Chen,

Beijing Institute of Ophthalmology, Beijing Tongren Hospital, Capital Medical University, Beijing 100730, China

Z. Wang

Beijing Tongren Eye Center, Beijing Tongren Hospital, Capital Medical University, Beijing 100730, China

1. mail: zhaokekewzy@hotmail.com

**EXPERIMENTAL SECTION**

**Preparation of materials**

Sodium hyaluronate (800–1000 kDa, cat. no. H293501), chitosan (100–200 mPa·s, cat. no. C105801), 4-(4,6-dimethoxy-1,3,5-triazin-2-yl)-4-methylmorpholinium chloride (DMTMM, 99%, cat. no. D110326), 3-aminophenylboronic acid (3-APBA, ≥98%, cat. no. A135767), and 4-carboxyphenylboronic acid (4-CPBA, ≥97%, cat. no. C101099) were acquired from Aladdin Biochemical Co., Ltd (Shanghai, China). Hydrochloric acid (HCl, 36.0-38.0%, cat. no. 10011008) was procured from Sinopharm Chemical Reagent Co., Ltd (Shanghai, China). Pluronic F-127 (PF127, cat. no. P2443-250G) was procured from Sigma-Aldrich (Saint Louis, MO, USA). Procyanidin C1 (PCC1, 99.79%, cat. no. HY-N2342) and Cy5 amine hydrochloride (Cy5, cat. no. HY-D1321) was acquired from MedChemExpress LLC (Princeton, NJ, USA). Calcein acetoxymethyl ester (AM) (cat. no. C2012) and propidium iodide (PI) (cat. no. ST511) were obtained from Beyotime Biotechnology Co., Ltd (Shanghai, China). The Cell Counting Kit-8 (CCK-8, cat. no. CK04) was acquired from DOJINDO Laboratories (Kawasaki, Japan). Phosphate-buffered saline (PBS, cat. no. C0221B) was procured from Gibco Beyotime (Shanghai, China). Hydrogels were heated to 37 °C to attain a solid state, thereafter undergoing fast freezing in liquid nitrogen. After freeze-drying, the hydrogels were sectioned into minute fragments for scanning electron microscopy imaging utilizing a Hitachi S-4800 (Hitachi, Tokyo, Japan). Fourier-transform infrared spectroscopy (FTIR) was conducted with a Thermo Scientific Nicolet iS5 spectrometer (Thermo, Wisconsin, USA). Nuclear magnetic resonance (NMR) spectra were obtained using a Bruker Avance III HD 400 MHz spectrometer (Billerica, MA, USA). Confocal laser scanning microscopy (CLSM) images were obtained utilizing a ZEISS LSM880 (ZEISS, Jena, Germany).

**Synthesis of injectable hydrogel.**

To manufacture phenylboronic acid-modified hyaluronic acid (PBAmHA), 40 mg of sodium hyaluronate and 100 mg of DMTMM were solubilized in 5 mL of PBS and agitated for 10 minutes. Subsequently, 20 mg of 3-APBA was included, and the mixture was incubated for 72 h at pH 7.0 to finalize the grafting reaction. The product underwent dialysis utilizing a dialysis tube (MWCO: 14,000; Union Carbide Corporation) against 1 L of deionized water for a duration of 3 days, with the water being replaced every 12 hours. The final product, PBAmHA, was acquired through freeze-drying and stored at 4 °C. Phenylboronic acid-modified chitosan (PBAmChi) was synthesized using a similar procedure, where 50 mg of sodium hyaluronate was replaced with chitosan, HCl was added to achieve a final concentration of 0.1 M, and 3-APBA was substituted with 4-CPBA. PF127 was utilized at 15 wt% for all hydrogels. The injectable thermosensitive hydrogel of PCC1/PHCF hydrogel was synthesized using a two-step method to form a physically crosslinked double-penetrating network. For example, to prepare the PCC1/PHCF-Gel-MC, the weight ratio of PBAmHA to PBAmChi was set at 2:1, with 30 mg of PBAmHA and 15 mg of PBAmChi dissolved in 8.5 mL of deionized water in a glass container, followed by the addition of PCC1 at concentrations of 750 μM (in vitro) and 1.5 mM (in vivo), followed by nitrogen purging to remove dissolved oxygen. The reaction was permitted to continue in darkness for 12 hours at 4 °C. Secondly, 1.5 g of PF127 was added and agitated overnight. Additional hydrogel formulations were synthesized using a similar procedure.

**Synthesis of PCC1-Cy5 and PCC1-Cy5@PHCF-Gel.**

To synthesize PCC1-Cy5, PCC1 (13 mg) was dissolved in TBS buffer (10 mL, pH 8.0) and stirred for 8 h. Cy5 (3 mg) was then added, and the reaction mixture was stirred for an additional 24 h in the dark. The crude product was purified by dialysis (MWCO 5,000) against deionized water for 24 h at 4 °C in the dark to remove unreacted PCC1 and free Cy5. PCC1-Cy5@PHCF-Gel was prepared by mixing PBAmHA and PBAmChi at a weight ratio of 2:1, with PCC1-Cy5 at a final concentration of 1.5 mM

**Rheology testing of the hydrogels.**

The sol-gel transition characteristics of the hydrogels were assessed utilizing a Discovery HR-2 rheometer (TA Instruments, DE, USA) equipped with a 25 mm steel plate. The strain amplitude and shear frequency were established at 0.1% and 1 Hz, respectively. Hydrogels were inserted between the plates with a 1 mm interval. The heating rate for temperature sweep tests was 1 °C min^-1^. Time sweep tests were performed on the T_gel_, which was obtained from the temperature sweep test to ascertain the gelation time.

Human primary retinal microvessel endothelial cells (HRMECs) were purchased from Cell Systems Corporation (Kirkland, WA, USA; cat. no. ACBRI 181, lot no. 123456) and cultured in endothelial cell medium (ScienCell; Carlsbad, CA, USA; cat. no. 1001) enriched with 5% fetal bovine serum, 1% ECGS, and 1% penicillin/streptomycin at 37 °C in a 5% CO_2_ incubator. Cells were routinely tested for mycoplasma contamination. HRMECs (passages 3-6) were seeded in 96-well plates (Corning, NY, USA) at 5,000 cells per well and cultured overnight. Experimental cells were randomly assigned to groups to ensure baseline consistency across groups. The medium was subsequently substituted with 100 μL of fresh medium incorporating diverse amounts of HA, chitosan, PHCF, or PCC1/PHCF-Gel-MC hydrogel. After 48 hours, the medium was substituted with 100 μL of fresh medium with 10% CCK-8 reagent, followed by a 2-hour incubation period. Cell viability was assessed by measuring absorbance at 450 nm with a microplate spectrophotometer Infinite F50 (TECAN, Männedorf, Switzerland), using untreated cells as the control. Each condition was evaluated in quintuplicate.

**Senescence modeling and cell viability analyses.**

We established a senescent HRMECs model by exposing cells to oxidative stress (150 μM H_2_O_2_ for 24 h at 37 °C), followed by culture in fresh medium for an additional 5 days. Cells were then treated with PCC1 at the indicated concentrations, and cell viability was assessed using the CCK-8 assay. Absorbance at 450 nm was measured using an Infinite F50 microplate reader (TECAN, Männedorf, Switzerland).

**Immunocytochemistry (ICC).**

Cells were fixed in 4% paraformaldehyde for 15 min and then blocked/permeabilized with 10% BSA containing 0.03% Triton X-100 for 60 min at room temperature. Cells were incubated with primary antibodies (Supplementary Table S1) overnight at 4 °C, washed three times with PBS, and then incubated with Alexa Fluor^®^ 488- or 594-conjugated secondary antibodies (anti-mouse or anti-rabbit; Invitrogen; 1:500) for 1 h at room temperature. Nuclei were counterstained with DAPI (Invitrogen; 1:5000) for 10 min.

**Flow cytometry.**

Apoptosis in senescent HRMECs was assessed using an apoptosis detection kit (Roche; cat. no. 11684795910) according to the manufacturer’s instructions. Senescent HRMECs were seeded in 10 cm dishes at a density of 1 × 10^6^ cells per dish and treated with PBS, PHCF-Gel, PCC1, or PCC1/PHCF-Gel for 48 h. Apoptotic cells were analyzed on a CytoFLEX flow cytometer (Beckman Coulter, Indianapolis, IN, USA), and data were processed using FlowJo v10.9.0. Apoptotic cells (Q3, Annexin V^+^/PI^−^; Q2, Annexin V^+^/PI^+^) were quantified.

**Animal study design and experimental models.**

All experimental procedures complied with the ARVO Statement for the Use of Animals in Ophthalmic and Vision Research and were approved by the Institutional Animal Care and Use Committee of Shanghai General Hospital, Shanghai Jiao Tong University School of Medicine (approval no. 2024AW042). Healthy adult Chinchilla rabbits (3 months old; 2.5–3.0 kg) were obtained from Jiagan Bio-Tech Co. and housed individually in stainless-steel cages under controlled conditions (18–22 °C; 50%–60% humidity; 12-h light/dark cycle). Rabbits were acclimatized for at least 7 days with ad libitum access to standard chow and water. Pregnant C57BL/6J mice for the oxygen-induced retinopathy (OIR) model were purchased from Vital River Laboratory Animal Technology Co., Ltd (Beijing, China). Adult male C57BL/6J mice (6–8 weeks old; 18–22 g) for laser-induced choroidal neovascularization (CNV) and in vivo imaging were obtained from SPF Biotechnology Co., Ltd (Beijing, China). Adult male BALB/c mice (6–8 weeks old; 25–30 g) for real-time fluorescence monitoring using an in vivo imaging system were obtained from BK/KY Biotechnology Co., Ltd (Shanghai, China). Mice were maintained in ventilated cages under a 12-h light/dark cycle at 23–25 °C and ~50% relative humidity. Animals were randomly assigned to treatment groups. Rabbits were euthanized by CO_2_ asphyxiation, and mice were euthanized by cervical dislocation. Eyeballs were harvested for retinal and choroidal tissue isolation, and blood and major organs were collected for subsequent analyses.

**Establishment of an OIR model.**

A hyperoxia chamber was purchased from Huaxi Electronics Technetronic Co., Ltd (Changsha, China). Pregnant C57BL/6J mice were monitored twice daily to determine the time of delivery, and the day of birth was designated as postnatal day 0 (P0). On P7, pups together with their nursing dams were placed in the chamber and exposed to 75% O_2_ from P7 to P12 (5 days) to induce retinal vaso-obliteration and suppress physiological angiogenesis. On P12, mice were returned to room air (normoxia). This model recapitulates early vascular loss (P7–P12), vascular regrowth (P12–P17), and pathological neovascularization (typically P14–P17), with peak neovascularization occurring at P17.^[2]^ Retinal vasculature was assessed by isolectin B4 (IB4, Invitrogen, cat. no. I21411) immunofluorescence (IF) imaging. At P17, OIR mice exhibited prominent avascular areas (AVAs) and neovascular tufts (NVTs) compared with normoxic controls, confirming successful model induction. Mice that did not meet the criteria for successful OIR induction were excluded from subsequent experiments. Neovascularization was quantified by counting preretinal nuclei on retinal cross-sections and by measuring AVAs and NVTs areas on retinal whole mounts using P.S. software (v26.2.0).

**Co-localization of SA-β-gal staining with IB4.**

Intracellular senescence was assessed using a senescence-associated β-galactosidase (SA-β-gal) staining kit (Beyotime, cat. no. C0602) according to the manufacturer’s instructions, with minor modifications for retinal tissue. Briefly, cells or retinas were washed twice with PBS and fixed with 4% formaldehyde (Thermo, cat. no. 28906) for 15 min (cells) or up to 2 h (retinas) at room temperature. After fixation, SA-β-gal staining was performed following the kit’s standard protocol for tissues. Because retinal flatmounts are more difficult to stain, samples were incubated in SA-β-gal staining solution at 37 °C in a non-CO_2_ incubator for at least 72 h. Retinal tissues were stained in 1.5-mL tubes (rather than in multi-well plates) and placed in a humidified chamber to prevent evaporation and drying during prolonged incubation. Successful staining was initially monitored by gross inspection; the appearance of an obvious blue-green coloration in the retina typically indicated positive SA-β-gal signal. If staining was weak, the incubation time was further extended until a stable color reaction was observed. Following SA-β-gal staining, retinal tissues were permeabilized and blocked in PBS containing 1% Triton X-100 and 5% BSA for 1 h at room temperature, followed by overnight incubation at 4 °C with IB4. Samples were subsequently counterstained with DAPI, mounted, and imaged. SA-β-gal signals were acquired by brightfield microscopy, whereas IB4 and DAPI signals were acquired by fluorescence microscopy from the same samples. Brightfield imaging for SA-β-gal staining was performed using an Olympus microscope equipped with appropriate optical filters (Olympus, Tokyo, Japan). Senescent cells and senescence-associated retinal regions were identified based on the presence of a characteristic blue-green precipitate under brightfield illumination. The area of blue-green staining in SA-β-gal-positive cells and retinas in sagittal sections, along with the staining area in flat-mounted retinas, was quantified using ImageJ (v 1.8.0.112), using established protocols.^[1]^

**RNA sequencing (RNA-seq) sample preparation and extraction methods.**

Retinas were harvested at P17 from normoxic and OIR mice. OIR mice received intravitreal injections of PBS or PHCF/PCC1-Gel at P15. Total RNA was extracted using TRIzol reagent (Invitrogen; cat. no. 15596018). RNA-seq libraries were prepared using the VAHTS Universal V10 RNA-seq Library Prep Kit (Premixed Edition; cat. no. NR616-02). Transcriptome sequencing and bioinformatic analysis were performed by Shanghai Ouyuan Biotechnology Co., Ltd. (Shanghai, China).

**RNA-seq and analysis.**

Libraries were sequenced on an Illumina NovaSeq X Plus platform to generate 2 × 150 bp paired-end reads. Each sample yielded approximately 6.0–7.2 million raw reads. Raw FASTQ files were quality-filtered using fastp to remove low-quality reads and generate clean reads for downstream analyses.^[3]^ Clean reads were aligned to the mouse reference genome using HISAT2,^[4,5]^ and gene-level read counts were obtained using HTSeq-count.^[6]^ Gene expression levels were additionally calculated as fragments per kilobase of transcript per million mapped reads (FPKM). Principal component analysis (PCA) and count-based visualizations were performed in R (v3.2.0) to assess the consistency of biological replicates. Differential gene expression analysis was performed using DESeq2, with genes meeting an adjusted P value (q-value) < 0.05 and fold change > 1.5 or < 0.5 defined as differentially expressed genes (DEGs).^[7]^ Gene set enrichment analysis (GSEA) was performed using gene sets curated in the Molecular Signatures Database (MSigDB), including KEGG gene sets (v7.2), and enrichment was evaluated using GSEA software.^[8,9]^ Genes were ranked by differential expression between groups, and enrichment of predefined gene sets was assessed at the extremes of the ranked list. RNA-seq data have been deposited in the GEO database under accession number GSE301407.

**Preparation of single-cell retinal suspension using 10x genomics** **technology**

Retinas were collected at P17 from OIR mice that received intravitreal PBS (n = 3) or PHCF/PCC1-Gel (n = 3) at P15. Under sterile conditions, freshly isolated retinas were washed twice with cold RPMI 1640 supplemented with 0.04% bovine serum albumin (BSA). Tissues were then minced into ~0.5 mm³ pieces using surgical scissors, incubated in freshly prepared digestion buffer at 37 °C for 15–30 min with gentle mixing every 5–10 min,^[10]^ and the digestion was stopped by adding cold medium. The digestion buffer contained RPMI 1640 (Gibco, cat. no. 11875093), 0.04% BSA (Sigma, cat. no. A1595), collagenase II (MCE, cat. no. HY-E70005B), trypsin (Generay, cat. no. GA0458), and DNase I (AppliChem, cat. no. A3778.0050).^[11]^ The resulting cell suspension was filtered through a 40-µm cell strainer (Falcon, cat. no. 352340) once or twice as needed, followed by centrifugation at 300 × g for 5 min at 4 °C. The pellet was resuspended in an appropriate volume of medium and washed twice (300 × g, 5 min each), with the supernatant discarded after each wash. Rod cells were isolated using PE Rat Anti-Mouse CD73 (TY/23) (BD, cat. no. 550741) and Goat Anti-Rat IgG (H+L) MicroBeads (Miltenyi, cat. no. 130-048-502) according to the manufacturer’s instructions.^[12]^ MACS BSA Stock Solution (Miltenyi, cat. no. 130-091-376) was diluted 1:20 in autoMACS Rinsing Solution (Miltenyi, cat. no. 130-091-222) to prepare the working buffer, and cells were resuspended in 100 μL of this buffer. Cell concentration and viability were assessed using a Luna-FL Cell Counter (Logos Biosystems, Gyeonggi-do, South Korea), and the single-cell suspension was adjusted to 700–1,200 cells μL^-1^. Single-cell library preparation and sequencing were performed according to the manufacturer’s instructions using the 10x Genomics Chromium Next GEM Single Cell 3′ Reagent Kits v3.1 (cat. no. PN-1000268). Libraries were sequenced on an Illumina NovaSeq X Plus platform.

**Analysis of 10× genomics single-cell RNA sequencing data.**

Library construction, sequencing, and data analysis were performed by OE Biotech Co., Ltd. (Shanghai, China). Cell Ranger (v8.0.1) with default parameters was used to align reads to the mouse reference genome (GRCm39), perform cell barcode filtering, and quantify gene expression. The filtered feature-barcode matrix was used for downstream analyses.^[13]^ Seurat (v4.0.0) was used for additional quality control to remove low-quality cells. Cells with <200 detected genes, <1,000 unique molecular identifiers (UMIs), log10GenesPerUMI <0.7, >5% mitochondrial UMIs, or >5% red blood cell gene expression were considered low quality and excluded. Doublets were identified and removed using DoubletFinder (v2.0.3).^[14]^ After quality control, data were normalized using the NormalizeData function in Seurat.

The FindVariableGenes function in Seurat (mean.function = FastExpMean, dispersion.function = FastLogVMR) was used to identify the top 2,000 highly variable genes (HVGs). PCA was performed using the HVG expression matrix, and batch effects were corrected using the RunHarmony function implemented in the harmony R package (v1.0).^[15,16]^ The integrated embeddings were visualized in two-dimensional space using uniform manifold approximation and projection (UMAP).

After clustering each sample, ClusterMap (v0.1.0) was run with default settings and a core value cutoff of 0.1 to compute Euclidean distances between cluster centroids and generate hierarchical clustering maps. A “senescence score” was calculated using the AddModuleScore function in Seurat based on the Fridman_Senescence_UP and Global_Senescence_Literature_Curated_2020 gene sets and visualized using ridge plots.^[17]^ Dot plots were generated using the DotPlot function in Seurat. Gene set files were obtained and curated using the GSEABase package (v1.44.0), including KEGG gene sets as well as SASP_Literature_Curated_UP and Global_Senescence_Literature_Curated_2020.^[12]^ Pathway activity scores at the single-cell level were quantified using GSVA (v1.30.0).^[9]^ Differential pathway activity between groups was assessed using limma (v3.38.3). The scRNA-seq data have been deposited in the GEO database under accession number GSE301406.

**Identification and validation of marker genes.**

Marker genes for endothelial Cluster 7 and microglial Cluster 6 were identified by differential expression analysis against all other clusters, followed by stringent dual-criterion filtering: (1) genes expressed in ≥60% of Cluster 7 cells with higher mean expression in Cluster 7, and (2) genes expressed in ≤30% of cells in all other clusters with lower mean expression outside Cluster 7. The top 10 candidate genes meeting these criteria were further prioritized based on prior literature supporting roles in cell-type-specific functions, senescence-associated pathways, and retina-relevant biology. The endothelial marker CXCR4 was validated by retinal flat-mount IF, whereas the microglial marker IFITM3 was assessed by IF staining of retinal cryosections (as described below). Images were obtained utilizing a confocal microscope (Celldiscoverer 7, Zeiss, Oberkochen, Germany).

**Laser-induced CNV model.**

The preoperative slit-lamp examination verified the lack of anterior segment or fundus abnormalities, and 1% tropicamide eye drops were administered to produce mydriasis. Mice were anesthetized with 50 mg mL^-1^ tiletamine hydrochloride and zolazepam hydrochloride (25 mg kg^-1^, intraperitoneally; Virbac, Zoletil 50) 5–10 minutes before the procedure. Levofloxacin ointment was administered to the corneal surface in contact with the coverslip. A 532 nm multi-wavelength laser photocoagulation apparatus (Coherent, Saxonburg, PA, USA) was employed to generate four laser spots encircling the optic nerve head at 1.5–2.0 optic disc diameters. The laser parameters were: wavelength of 532 nm; power of 120 mW; spot diameter of 50 μm; and exposure duration of 100 ms. Laser spots were strategically placed to circumvent significant retinal vessels. Successful rupture of Bruch’s membrane was verified by the bubble development after photocoagulation. Mice exhibiting failed membrane rupture or retinal hemorrhage at the laser location were excluded from further analysis.

**Comprehensive imaging protocol for the assessment of CNV.**

The animal CNV model was assessed using a comprehensive imaging approach. Mice were given 1% tropicamide ocular drops to produce mydriasis. Upon achieving sufficient pupil dilation, general anesthesia was achieved with an intraperitoneal injection of 50 mg mL^-1^ tiletamine hydrochloride and zolazepam hydrochloride, and topical anesthesia was utilized with 0.5% oxybuprocaine hydrochloride (Santen Pharmaceuticals, Osaka, Japan). Fluorescein fundus angiography (FFA), fundus photography (FP), and optical coherence tomography (OCT) were subsequently conducted utilizing the Micron IV System (Phoenix Research Laboratories, Pleasanton, CA, USA). FFA imaging was initially employed to evaluate vascular leakage associated with CNV. A 50 µL intraperitoneal injection of 10% sodium fluorescein solution (Alcon, cat. no. 0065-0092-65) was provided during FFA. The diagnosis of CNV was predicated on fluorescence leakage observed during the early (within 5 minutes) and late (after 10 minutes) phases of angiography. Typically, CNV exhibits the following features: (1) early manifestation during the arterial or pre-arterial phase; (2) leakage sites that are independent of retinal vasculature; and (3) fast fluorescein leakage with a defined accumulation zone, leading to a hyperfluorescent region in the late phase. Two separate specialists evaluated the grades of CNV leakage utilizing late-phase hyperfluorescence: Grade I exhibited no hyperfluorescence; Grade II indicated hyperfluorescence without leakage; Grade III presented early or mid-phase hyperfluorescence with late leakage; and Grade IV demonstrated intense hyperfluorescence that augmented in both size and intensity during the transit phase, deemed clinically significant. Animals graded as IV were chosen for additional analysis. OCT imaging was utilized to acquire cross-sectional profiles of the lesion, while FP was performed to evaluate CNV lesion dimensions.

**Intravitreal injections.**

Prior to the procedure, all animals were administered 1% tropicamide eye drops to induce mydriasis. Chinchilla rabbits were sedated with isoflurane, and a lid speculum was employed to expose the eyeball completely. A corneal contact plano lens was utilized to enhance the visibility of the injected gel. C57BL/6J and BALB/c mice (aged 6–8 weeks) were sedated via an intraperitoneal injection of 50 mg mL^-1^ tiletamine hydrochloride and zolazepam hydrochloride, 5–10 minutes before the procedure. Subsequently, 0.5% oxybuprocaine hydrochloride was administered for topical anesthesia. Mice were placed laterally to ensure complete exposure of the eyeball. A 33G Hamilton syringe (Hamilton, Bonaduz, Switzerland) was employed to puncture the vitreous cavity at a 30° angle, approximately 1 mm below the limbus, to a depth of 1–2 mm. A microinjection pump was subsequently employed to gradually inject 1 μL of PBS, Aflibercept, PCC1, PCC1/PHCF-Gel. The needle was maintained in position for 10 seconds before extraction to avert backflow. General anesthesia was unnecessary for P15 pups because of their thin sclera and diminutive stature. Following the application of topical anesthesia with 0.5% oxybuprocaine hydrochloride, an intravitreal injection was executed using a sharp 33G Hamilton syringe. Levofloxacin ointment was immediately administered following the injection to avert infection. Animals exhibiting substantial reflux, intraocular bleeding, or unintentional lens perforation were omitted from further analysis.

**Electroretinography (ERG).**

ERG was conducted for OIR and CNV model animals at P17 and P14, respectively. As previously detailed, full-field ERGs were recorded utilizing the RETIport System (Roland Consult, Brandenburg, Germany) in conjunction with a Super Color Ganzfeld (Q450 SC) stimulator.^[18]^ The mice underwent overnight dark adaptation before the procedure. Anesthesia was administered with an intraperitoneal injection of 50 mg mL^-1^ tiletamine hydrochloride and zolazepam hydrochloride, and pupil dilation was accomplished with 1% tropicamide. A heating pad was employed to sustain body temperature at 37 °C during the process. Reference and ground electrodes were positioned at the midline of the scalp and the upper tail skin, respectively. Following the application of topical anesthesia with 0.5% oxybuprocaine hydrochloride, contact lens electrodes were placed on the corneal surface. Mice were aligned with the center of the Ganzfeld bowl to provide consistent illumination of both eyes. All procedures were conducted under subdued red light. Scotopic ERG responses were obtained at a light intensity of 3.0 cd·s·m^-2^. Following a 10-minute light adaptation period, photopic ERG responses were documented under the same intensity. Finally, the 30 Hz flicker ERG was conducted. The amplitude of the ERG a-wave was quantified from the baseline to the trough of the initial negative deflection, whereas the b-wave amplitude was assessed from the a-wave trough to the peak of the b-wave. All amplitude measurements were computed automatically.

**Mouse cardiac perfusion.**

Mice were positioned supinely and affixed to a foam board. A surgical incision was performed 2–3 mm to the left of the sternum to access the thoracic cavity. The heart was revealed through meticulous dissection of the rib cage. An incision was created in the right atrium with ophthalmic scissors, and a 23-G scalp needle was introduced into the left ventricle at a depth that prevented perforation. A peristaltic pump was employed to perfuse the animal with newly produced 0.9% NaCl (saline). Perfusion was deemed successful when the effluent lacked blood, and muscular contractions were observed in the limbs and tail. The procedure ended when the animal exhibited rigidity and the liver appeared pallid.

**Retinal flat mount and IF.**

Following cardiac perfusion, the enucleated eye was promptly immersed in 4% formaldehyde and preserved for 1 hour at room temperature. The eye was thereafter cleaned two to three times with 1× PBS. A minor incision was executed in the cornea with scissors, and the sclera was delicately retracted toward the optic nerve using forceps. The retina was isolated by excising the cornea, lens, iris, sclera, optic nerve, and retinal pigment epithelium. Any debris, loose vessels, and vitreous remnants were removed from the retinal cup using forceps. The retina was permeabilized and blocked for 1 hour at room temperature on a shaker with 5% BSA and 1% Triton X-100, after three PBS washes. The retina was treated overnight at 4 °C on a shaker in a primary antibody dilution solution (5% BSA + 1% Triton X-100), as detailed in Supplementary Table 1. Secondary antibodies conjugated to Alexa Fluor^®^ 488/555/594/647 (1:500, Invitrogen), specific for mouse/rabbit/goat/guinea pig IgG were incubated for 1 hour at room temperature. Following three PBS washes, the DAPI working solution was applied and incubated for 5–8 minutes at room temperature. The retina was affixed on a glass slide with the photoreceptor side facing downward and stabilized to prevent movement. Four radial incisions were executed 1 mm from the optic nerve to the peripheral retina, segmenting it into four equal quadrants. Each quadrant was compressed utilizing forceps. A drop of mounting medium containing antifade reagent (Sigma, cat. no. [P36930](https://www.thermofisher.cn/order/catalog/product/P36930)) was applied prior to carefully placing a coverslip to minimize air bubbles and ensure adequate flattening. Images were obtained utilizing a confocal microscope (Celldiscoverer 7, Zeiss, Oberkochen, Germany).

**Choroidal-retinal flat mount and IF.**

Eyes were meticulously enucleated after perfusion to prevent mechanical injury. The harvested eyes were immersed in 4% formaldehyde for 1 hour at room temperature, thereafter undergoing three PBS washes. Utilizing a microscope, the eyes were bisected at the equator, the anterior segment was excised, and the retinal nerve fiber layer was meticulously detached to isolate the RPE-choroid-sclera complex. The complexes were subsequently blocked and permeabilized in a solution of 5% BSA and 0.3% Triton X-100 for 1 hour at room temperature. The tissues were incubated overnight at 4 °C with primary antibodies, followed by washing. On the subsequent day, following three PBS washes, the tissues were incubated for 1 hour at room temperature with Alexa Fluor 488/555/594/647-conjugated secondary antibodies against mouse, rabbit, goat, or guinea pig IgG. Following three additional PBS washes, the DAPI working solution was formulated and administered for 5–8 minutes at room temperature. The RPE-choroid-sclera complexes were subsequently cleaned, incised radially in four directions to facilitate flattening, affixed on glass slides, and covered with a coverslip after applying an anti-fade reagent. Images were obtained utilizing a Zeiss Celldiscoverer 7 confocal microscope (Zeiss, Oberkochen, Germany).

**Cryosectioning and IF.**

After perfusion, model mice were initially fixed in 4% formaldehyde for 5 minutes at room temperature. The anterior segment was excised, and the tissues were further fixed for 30 minutes before immersion in PBS. With microscopic assistance, the anterior segment and lens were meticulously excised, preserving the integrity of the limbus and preventing retinal detachment. The ocular globes were sequentially dehydrated in 15% and 30% sucrose solutions for 30 minutes each at room temperature. Dehydration was deemed complete when the eyes noticeably retracted inward. An aluminum lid was pre-cooled in liquid nitrogen, and the optimal cutting temperature compound (Fisher Scientific, cat. no. 23-730-571) embedded eye blocks were swiftly frozen on its surface, avoiding direct immersion in liquid nitrogen to prevent cracking. Samples were preserved at -80 °C or immediately underwent cryosectioning. For IF, sections were incubated in PBS with 5% BSA and 0.3% Triton X-100 for 1 hour. Subsequently, primary antibodies (detailed in Supplementary Table S1) were administered. The following day, sections were treated with Alexa Fluor 488/594/647-conjugated secondary antibodies (1:500). Following three washes with PBS, the DAPI working solution was administered for 5–8 minutes at room temperature. Images were acquired utilizing the Zeiss Celldiscoverer 7 confocal microscope (Zeiss, Oberkochen, Germany).

**DHE and DCF staining.**

To evaluate OIR and superoxide levels, unfixed retinal cryosections were stained using 2′, 7′-dichlorofluorescin diacetate (DCF; Sigma, cat. no. D6883) and dihydroethidium (DHE; Fisher Scientific, cat. no. D11347). Mouse eyes were recently enucleated, implanted in optimal cutting temperature compound, and promptly snap-frozen on dry ice. Sections (12 μm thick) were produced and maintained frozen throughout the procedure. The slides were immersed in ice-cold acetone for 10 minutes at -20 °C, allowed to equilibrate to room temperature for 20 minutes, and washed three times in PBS for 5 minutes each. The sections were incubated at room temperature in the dark with 10 μM DCF or 0.625 μM DHE for 60 minutes or 20 minutes, respectively.^[19]^ After incubation, the slides were rinsed thrice in PBS (5 minutes each), mounted with DAPI Fluoromount-G (SouthernBiotech, cat. no. 0100-20), and photographed with a Zeiss Celldiscoverer 7 confocal microscope.

**TUNEL staining.**

The Apoptosis Detection Kit (Roche, cat. no. 11684795910) was utilized to identify apoptosis. Cryosections of ocular tissue were initially frozen in an eyeball fixation solution for 15 minutes, and then underwent three PBS washes. The sections were subsequently permeabilized using freshly prepared 0.1% Triton X-100. Primary antibodies were administered and incubated overnight at 4 °C. The following day, secondary antibodies were incubated at room temperature for 1 hour. TUNEL labeling was conducted by incubating the sections with terminal deoxynucleotidyl transferase solution at 37 °C for 60 minutes. The sections were ultimately counterstained with DAPI and visualized using a Zeiss Celldiscoverer 7 confocal microscope (Zeiss, Oberkochen, Germany).

**Hematoxylin and eosin (H&E) staining.**

Eyeballs from each experimental group were preserved in FAS fixation solution (Servicebio, cat. no. G1109-50ML) and subsequently processed into paraffin-embedded slices. Regions with the most significant lesions were chosen for examination. The sections were deparaffinized and stained with H&E using conventional procedures.

**In vivo fluorescence imaging (IVFI).**

To assess the intraocular delivery efficiency of PCC1/PHCF-Gel, two mouse strains, C57BL/6J and BALB/c, were employed. Before imaging, all mice were administered 1% tropicamide eye drops to induce mydriasis and received an injection of PCC1/PHCF-Gel. Anesthesia was administered using isoflurane, and the mice were positioned on the imaging platform with their eyes uncovered. The ophthalmic gel was utilized to preserve corneal moisture. The imaging device was configured to an excitation wavelength of 640 nm for Cy5, and the ocular region was scanned for fluorescence signal detection. Pharmacokinetic analyses were conducted between free Cy5-labeled PCC1 (PCC1-Cy5) and hydrogel-encapsulated PCC1-Cy5 (PCC1-Cy5@PHCF-Gel) following intravitreal injection. Real-time fluorescence imaging was conducted with the Vieworks IVIS Smart LF system (Gyeonggi Province, South Korea) at 0, 3, 7, 14, 21, 28 and 35 days post-injection.

**Statistical analysis.**

Statistical analyses were performed using GraphPad Prism 10.0 (GraphPad Software, USA). All data are presented as mean ± SEM, and n denotes the number of biologically independent samples. For comparisons between two groups, an unpaired two-tailed Student’s t-test was used to determine p-values. For comparisons among more than two groups, one-way ANOVA was performed, followed by Tukey’s post hoc test as appropriate (specified in the corresponding figure legends). For experiments involving two independent variables, two-way ANOVA followed by Bonferroni’s test was used. No data points were excluded unless explicitly stated. Statistical significance levels were established as follows: **p* < 0.05, ***p* < 0.01, ****p* < 0.001, and *****p* < 0.0001. “ns” denotes no significant difference. Additional statistical details are available in the figure legends.

**References**

[1] M. Oubaha, K. Miloudi, A. Dejda, V. Guber, G. Mawambo, M.-A. Germain, G. Bourdel, N. Popovic, F. A. Rezende, R. J. Kaufman, F. A. Mallette, P. Sapieha, *Sci. Transl. Med.* **2016**, *8*, 362ra144.

[2] K. M. Connor, N. M. Krah, R. J. Dennison, C. M. Aderman, J. Chen, K. I. Guerin, P. Sapieha, A. Stahl, K. L. Willett, L. E. H. Smith, *Nat. Protoc.* **2009**, *4*, 1565.

[3] S. Chen, Y. Zhou, Y. Chen, J. Gu, *Bioinformatics* **2018**, *34*, i884.

[4] A. Roberts, C. Trapnell, J. Donaghey, J. L. Rinn, L. Pachter, *Genome Biol.* **2011**, *12*, R22.

[5] D. Kim, B. Langmead, S. L. Salzberg, *Nat. Methods* **2015**, *12*, 357.

[6] S. Anders, P. T. Pyl, W. Huber, *Bioinformatics* **2015**, *31*, 166.

[7] M. I. Love, W. Huber, S. Anders, *Genome Biol.* **2014**, *15*, 550.

[8] V. K. Mootha, C. M. Lindgren, K.-F. Eriksson, A. Subramanian, S. Sihag, J. Lehar, P. Puigserver, E. Carlsson, M. Ridderstråle, E. Laurila, N. Houstis, M. J. Daly, N. Patterson, J. P. Mesirov, T. R. Golub, P. Tamayo, B. Spiegelman, E. S. Lander, J. N. Hirschhorn, D. Altshuler, L. C. Groop, *Nat. Genet.* **2003**, *34*, 267.

[9] A. Subramanian, P. Tamayo, V. K. Mootha, S. Mukherjee, B. L. Ebert, M. A. Gillette, A. Paulovich, S. L. Pomeroy, T. R. Golub, E. S. Lander, J. P. Mesirov, *Proc. Natl. Acad. Sci.* **2005**, *102*, 15545.

[10] B. S. Clark, G. L. Stein-O’Brien, F. Shiau, G. H. Cannon, E. Davis-Marcisak, T. Sherman, C. P. Santiago, T. V. Hoang, F. Rajaii, R. E. James-Esposito, R. M. Gronostajski, E. J. Fertig, L. A. Goff, S. Blackshaw, *Neuron* **2019**, *102*, 1111.

[11] I. Benhar, J. Ding, W. Yan, I. E. Whitney, A. Jacobi, M. Sud, G. Burgin, K. Shekhar, N. M. Tran, C. Wang, Z. He, J. R. Sanes, A. Regev, *Nat. Immunol.* **2023**, *24*, 700.

[12] F. Binet, G. Cagnone, S. Crespo-Garcia, M. Hata, M. Neault, A. Dejda, A. M. Wilson, M. Buscarlet, G. T. Mawambo, J. P. Howard, R. Diaz-Marin, C. Parinot, V. Guber, F. Pilon, R. Juneau, R. Laflamme, C. Sawchyn, K. Boulay, S. Leclerc, A. Abu-Thuraia, J.-F. Côté, G. Andelfinger, F. A. Rezende, F. Sennlaub, J.-S. Joyal, F. A. Mallette, P. Sapieha, *Science* **2020**, *369*, eaay5356.

[13] Y. Hao, S. Hao, E. Andersen-Nissen, W. M. Mauck, S. Zheng, A. Butler, M. J. Lee, A. J. Wilk, C. Darby, M. Zager, P. Hoffman, M. Stoeckius, E. Papalexi, E. P. Mimitou, J. Jain, A. Srivastava, T. Stuart, L. M. Fleming, B. Yeung, A. J. Rogers, J. M. McElrath, C. A. Blish, R. Gottardo, P. Smibert, R. Satija, *Cell* **2021**, *184*, 3573.

[14] C. S. McGinnis, L. M. Murrow, Z. J. Gartner, *Cell Syst.* **2019**, *8*, 329.

[15] V. Lambert, J. Lecomte, S. Hansen, S. Blacher, M.-L. A. Gonzalez, I. Struman, N. E. Sounni, E. Rozet, P. De Tullio, J. M. Foidart, J.-M. Rakic, A. Noel, *Nat. Protoc.* **2013**, *8*, 2197.

[16] I. Korsunsky, N. Millard, J. Fan, K. Slowikowski, F. Zhang, K. Wei, Y. Baglaenko, M. Brenner, P. Loh, S. Raychaudhuri, *Nat. Methods* **2019**, *16*, 1289.

[17] S. Crespo-Garcia, P. R. Tsuruda, A. Dejda, R. D. Ryan, F. Fournier, S. Y. Chaney, F. Pilon, T. Dogan, G. Cagnone, P. Patel, M. Buscarlet, S. Dasgupta, G. Girouard, S. R. Rao, A. M. Wilson, R. O’Brien, R. Juneau, V. Guber, A. Dubrac, C. Beausejour, S. Armstrong, F. A. Mallette, C. B. Yohn, J.-S. Joyal, D. Marquess, P. J. Beltran, P. Sapieha, *Cell Metab.* **2021**, *33*, 818.

[18] S. Chen, J. Zhang, D. Sun, Y. Wu, J. Fang, X. Wan, S. Li, S. Zhang, Q. Gu, Q. Shao, J. Dong, X. Xu, F. Wei, Q. Sun, *Investig. Opthalmology Vis. Sci.* **2023**, *64*, 8.

[19] H. Liu, N. A. Stepicheva, S. Ghosh, P. Shang, O. Chowdhury, R. A. Daley, M. Yazdankhah, U. Gupta, S. L. Hose, M. Valapala, C. S. Fitting, A. Strizhakova, Y. Shan, D. Feenstra, J.-A. Sahel, A. Jayagopal, J. T. Handa, J. S. Zigler, P. E. Fort, A. Sodhi, D. Sinha, *Nat. Commun.* **2022**, *13*, 6045.


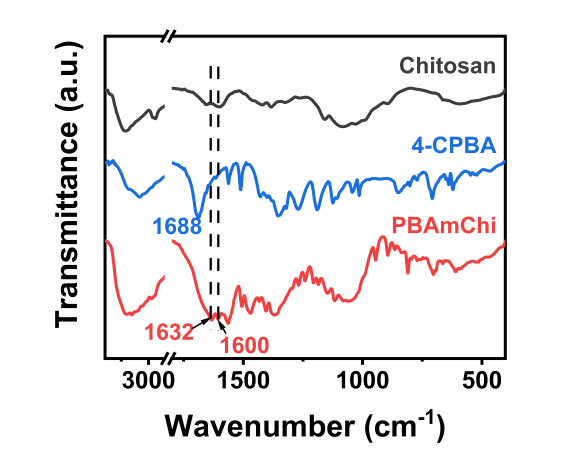


**Figure S1.** FTIR analyses of chitosan, 4-CPBA, and PBAmChi.


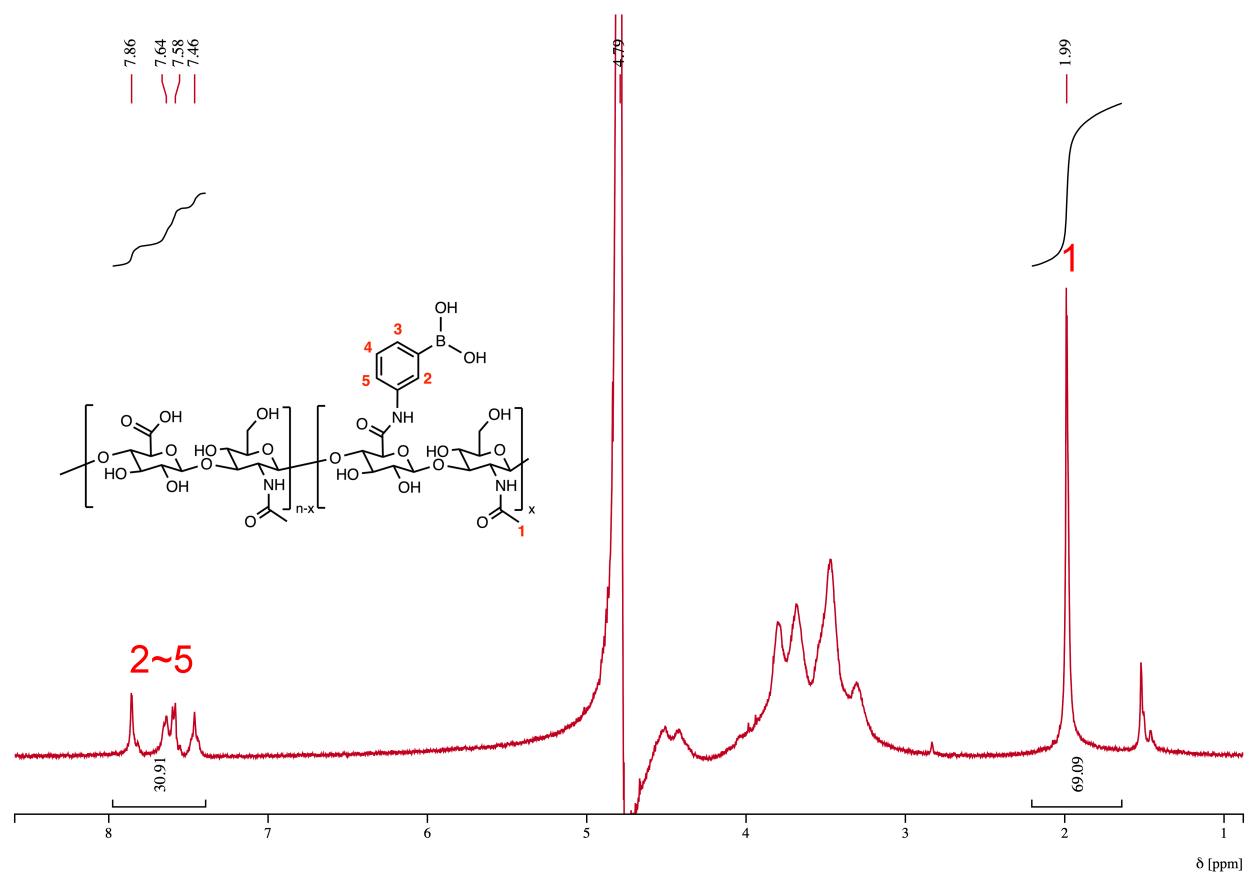


**Figure S2.** NMR spectrum of PBAmHA.


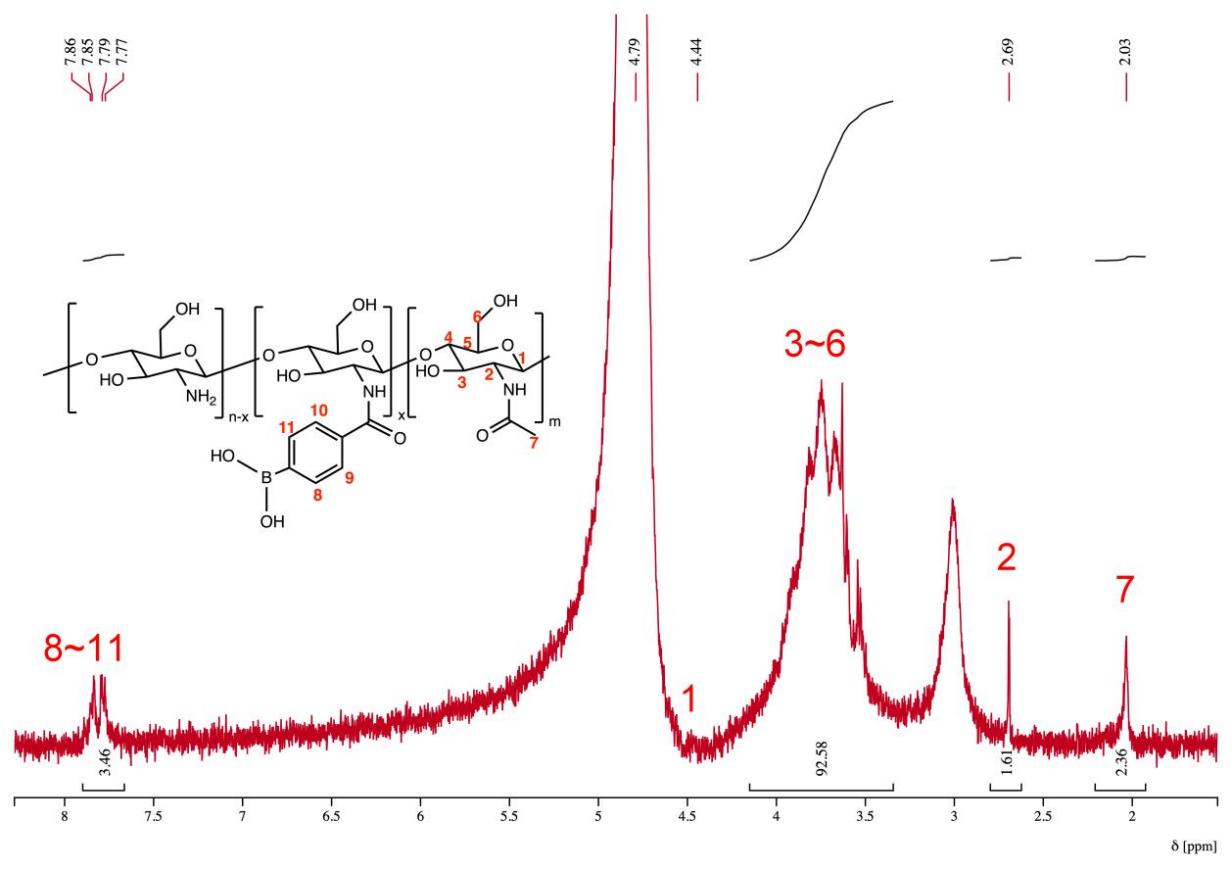


**Figure S3.** NMR spectrum of PBAmChi.


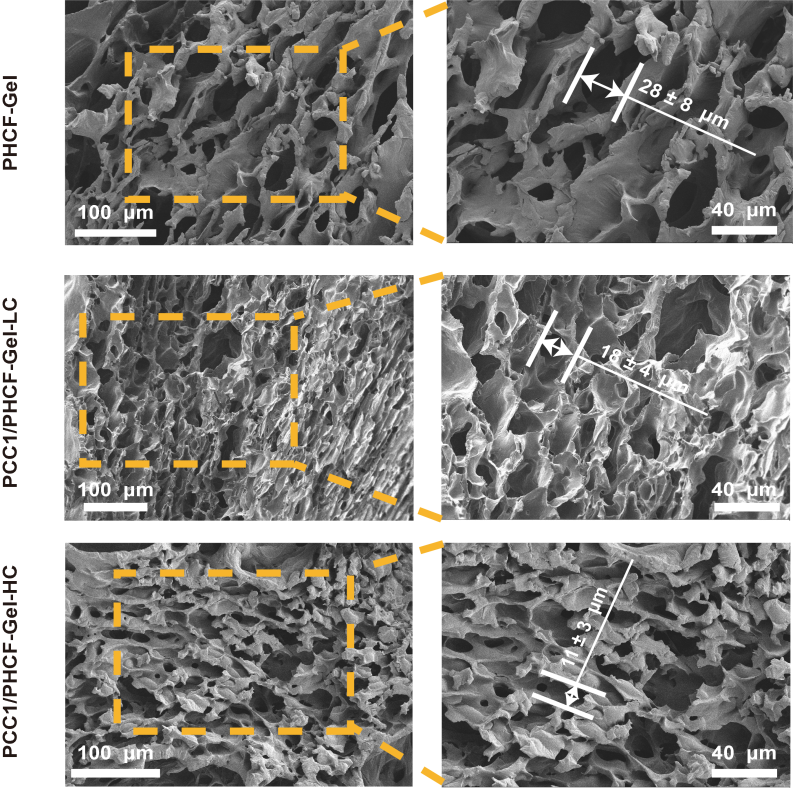


**Figure S4.** Scanning electron microscopy images of PHCF-Gel, PCC1/PHCF-Gel-LC, and PCC1/PHCF-Gel-HC.


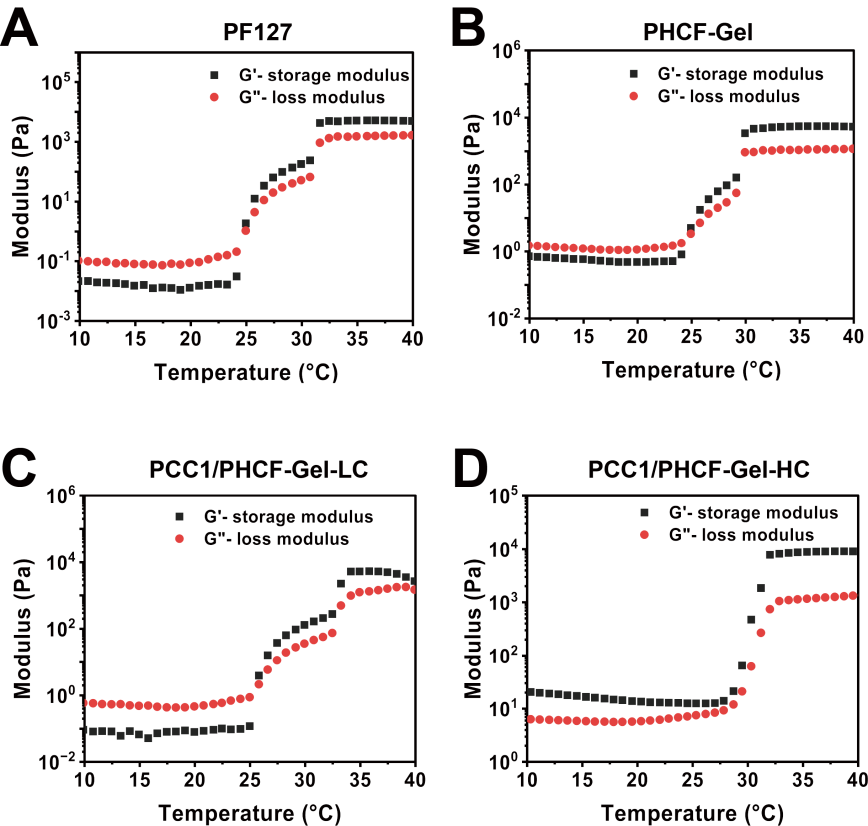


**Figure S5.** Temperature-dependent G′ and G′′ of (A) PF127, (B) PHCF-Gel, (C) PCC1/PHCF-Gel-LC, and (D) PCC1/PHCF-Gel-HC preparations.

**
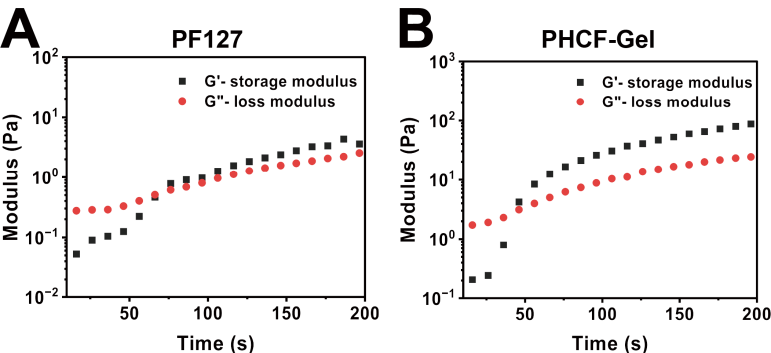
**

**Figure S6.** Time-dependent G′ and G′′ of (A) PF127 and (B) PHCF-Gel preparations. Experiments were repeated three times.


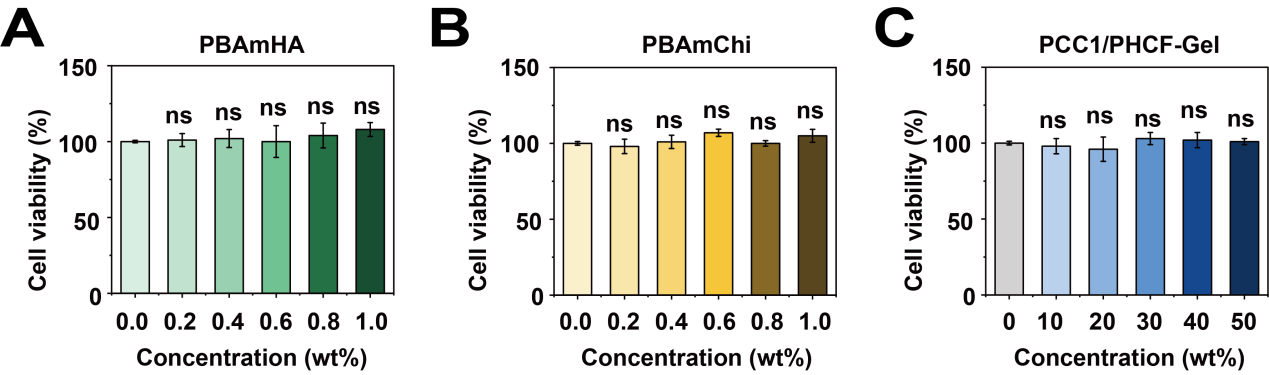
**Figure S7.** Viability of HRMECs cocultured with (A) PBAmHA, (B) PBAmChi, and (C) PCC1/PHCF-Gel. Data are presented as the mean ± SEM (n = 6). The p-values were calculated using one-way ANOVAs with Tukey’s post-test. ns, not significance.


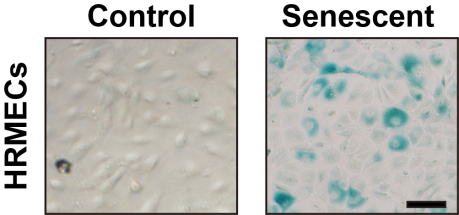


**Figure S8.** Senescent HRMECs model was validated by SA-β-gal staining, which resulted in a blue-green coloration in contrast to control cells. Scale bar, 100 μm.


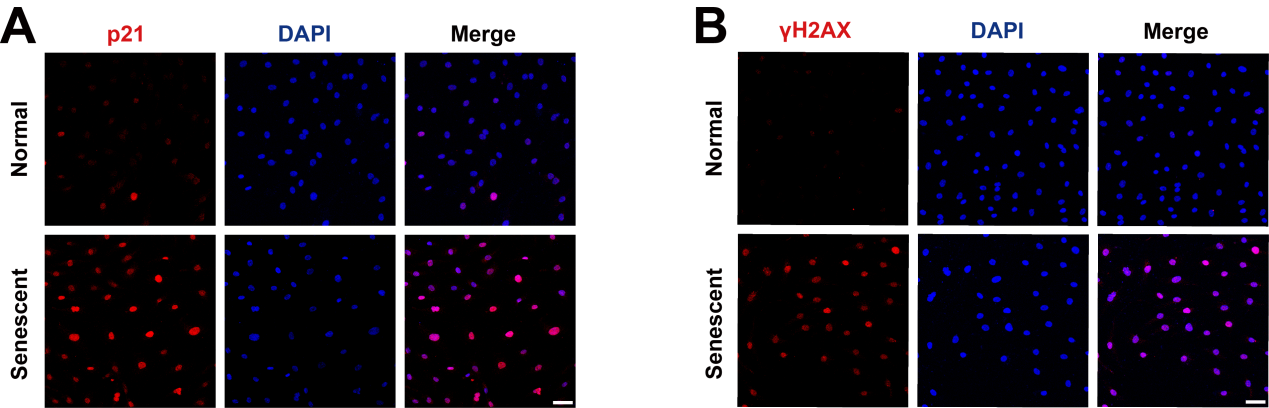


**Figure S9.** (A, B) Comparison of the expression of the senescence markers p21 and γH2AX between senescent HRMECs and normal cells. Scale bars, 50 µm.


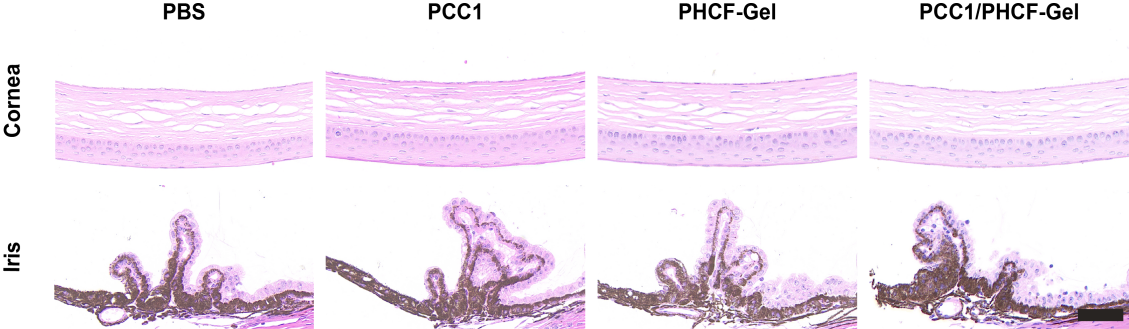


**Figure S10.** Representative images of H&E-stained sections of ocular tissues (cornea and iris) from mice that received intravitreal injections of PBS, PCC1, PHCF-Gel, and PCC1/PHCF-Gel. The mice were evaluated 1 month post-injection (n = 3). Scale bar, 50 μm.

**
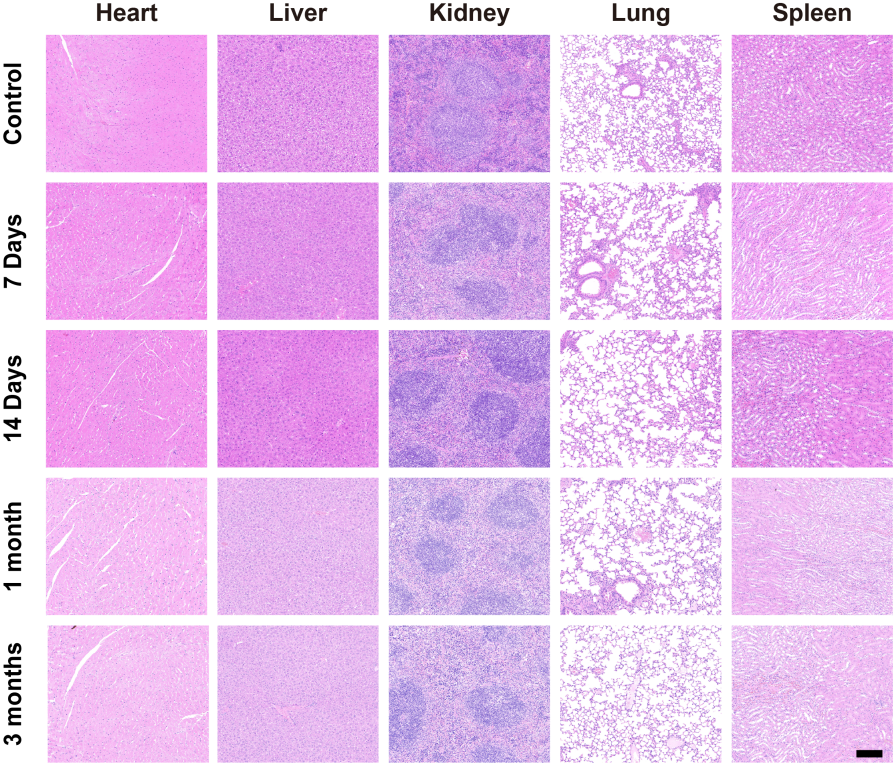
**

**Figure S11.** Representative H&E-stained sections of the major organs (heart, liver, spleen, lungs, and kidneys) from mice that received intravitreal injections of PCC1/PHCF-Gel. Analyses were conducted on the 7th day, 14th day, first month, and third month post-injection. Control mice were administered PBS (n = 3). Scale bar, 200 μm.


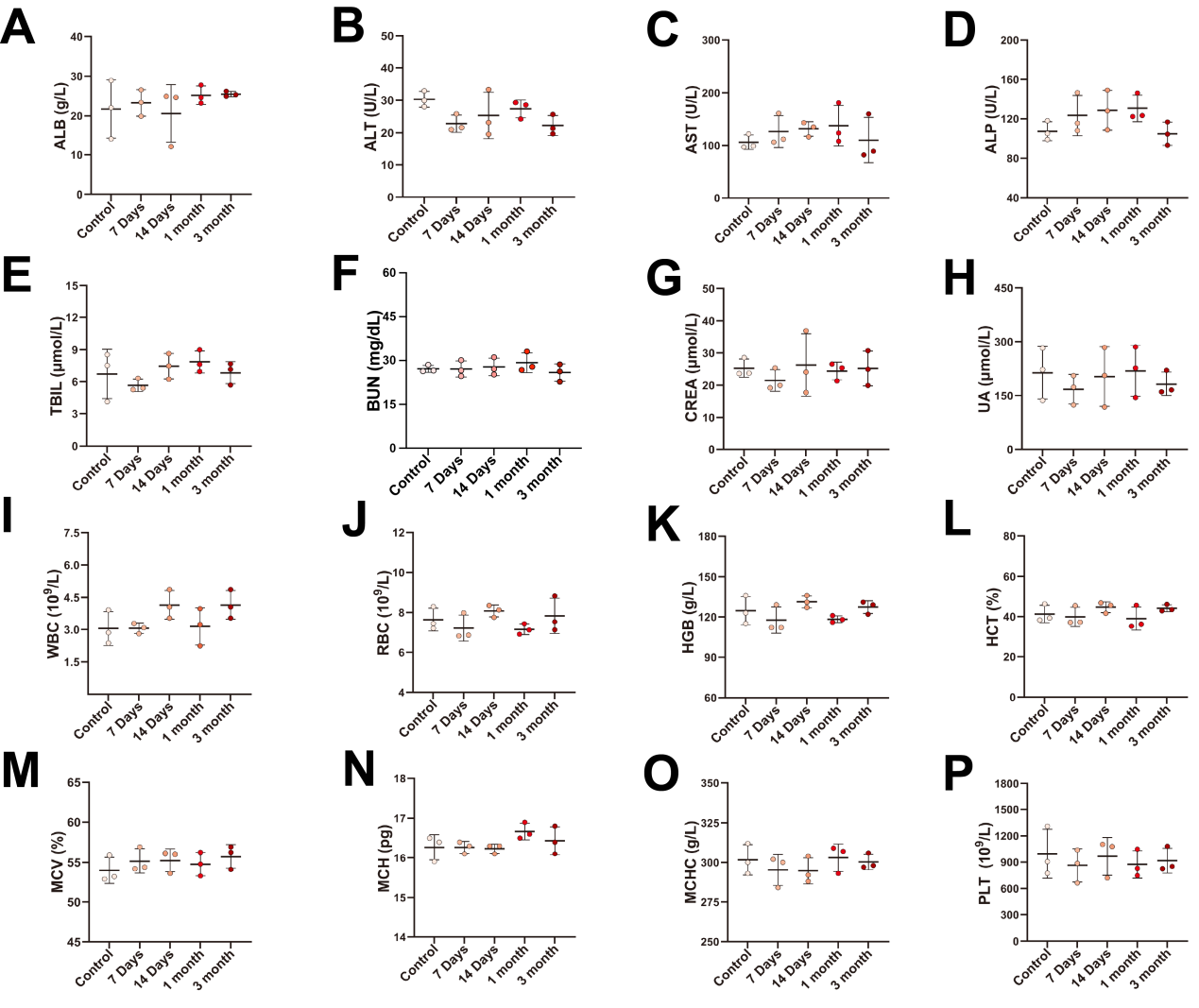


**Figure S12.** Blood biochemistry and comprehensive blood analyses of mice subjected to the intravitreal injection of PCC1/PHCF-Gel at multiple points (the 7th day, 14th day, first month, and third month post-injection). The evaluated parameters included the following: (A) albumin (ALB); (B) alanine aminotransferase (ALT); (C) aspartate aminotransferase (AST); (D) alkaline phosphatase (ALP); (E) total bilirubin (TBIL); (F) blood urea nitrogen (BUN); (G) creatinine (CREA); (H) uric acid (UA); (I) white blood cell (WBC) count; (J) red blood cell (RBC) count; (K) hemoglobin (HGB); (L) hematocrit (HCT); (M) mean corpuscular volume (MCV); (N) mean corpuscular hemoglobin (MCH); (O) mean corpuscular hemoglobin concentration (MCHC); and (P) platelet (PLT) count. Data are presented as the mean ± SEM (n = 3).


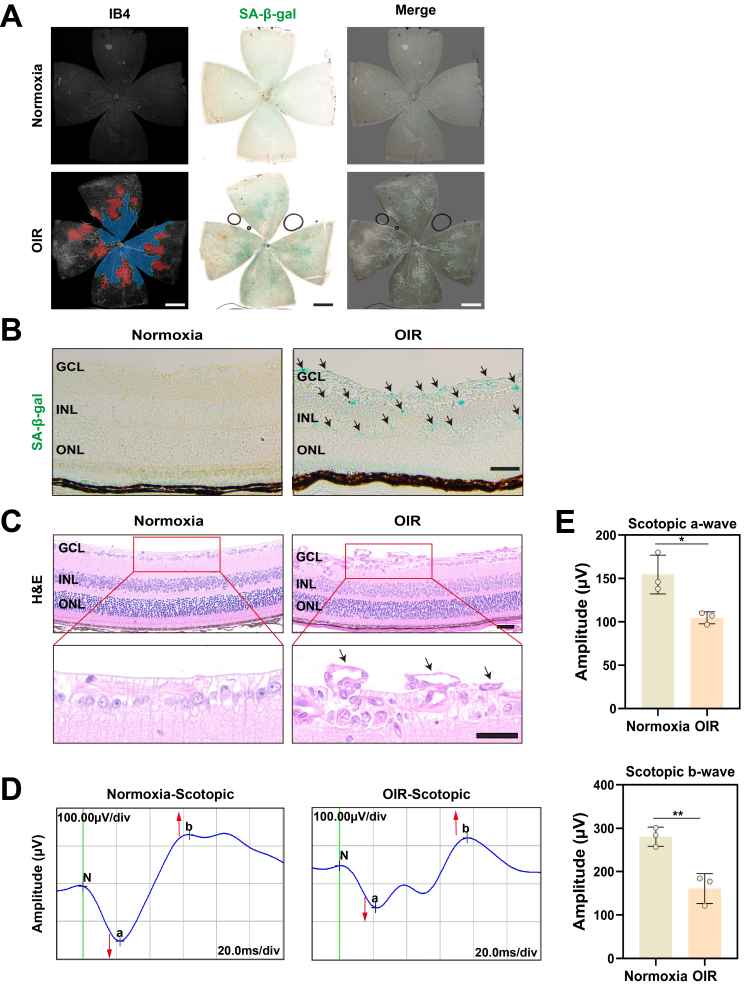


**Figure S13.** (A) Representative microscopy images of P17 OIR flat-mounted retinas compared with normoxic mice: IB4 staining (red zone for NVTs and blue zone for AVAs), SA-β-gal staining (blue-green), and co-localization of IB4 staining with SA-β-gal staining. (B) SA-β-gal staining of retinal tissue sections reveals the presence of senescence in the vasculature (indicated by black arrows) within the OIR model compared with normoxic mice (GCL, ganglion cell layer; INL, inner nuclear layer, and ONL, outer nuclear layer). (C) Representative H&E images were used to illustrate neovascular nuclei (indicated by black arrows) in OIR retinal tissue sections compared with normoxic mice. (D) Representative ERG wave responses of normoxic mice and OIR mice at P17 under scotopic conditions, along with measurement of these waves (E). Three independent experiments were performed for data analysis in vivo. Data are presented as the mean ± SEM (n = 3). The p-values were calculated using Student’s t-test. *p < 0.05 and **p < 0.01. Scale bars, 1 mm in (A) and 50 μm in (B,C).
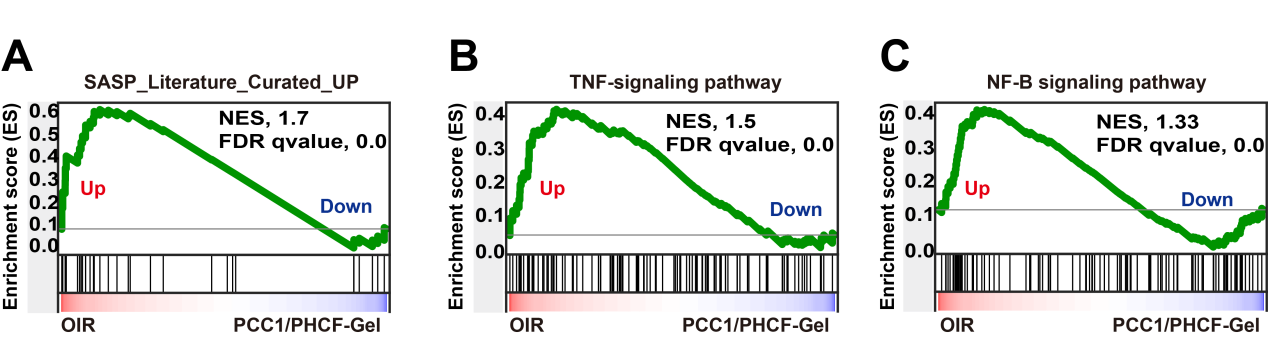


**Figure S14.** (A–C) GSEA analysis comparing PBS (n = 2) and PCC1/PHCF-Gel treatments (n = 3) based on bulk RNA-seq data from the P17 OIR model. The analysis utilized the SASP_Literature_Curated_UP, TNF, and NF-κB pathways gene sets. NES, normalized enrichment score; FDR, false discovery rate.


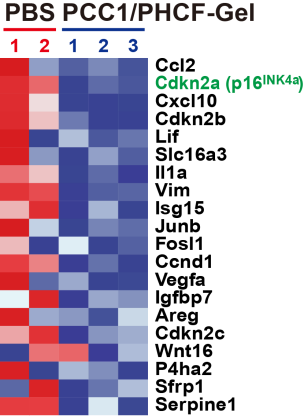


**Figure S15.** The heatmap highlights the 20 most enriched differentially expressed genes from the Global_Senescence_Literature_Curated_2020 gene set, based on bulk RNA-seq data comparing PBS (n=2) and PCC1/PHCF-Gel treatment (n=3).


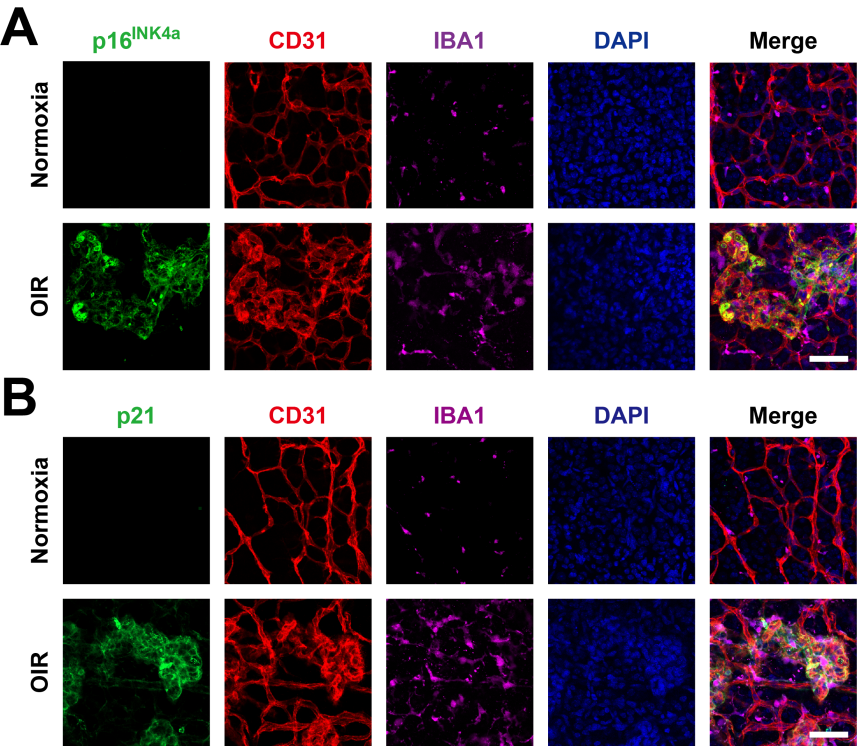


**Figure S16.** (A, B) Representative confocal IF images depicting the senescence markers p16^INK4a^ (A, green) and p21 (B, green), the vascular marker CD31 (red), the microglial marker IBA1 (purple), and DAPI (a nuclear marker, blue) in normoxic and OIR mice in vivo. The yellow color signifies senescent vascular endothelial cells, whereas white color indicate senescent microglial cells. Experiments were repeated three times. Scale bars, 50 μm.


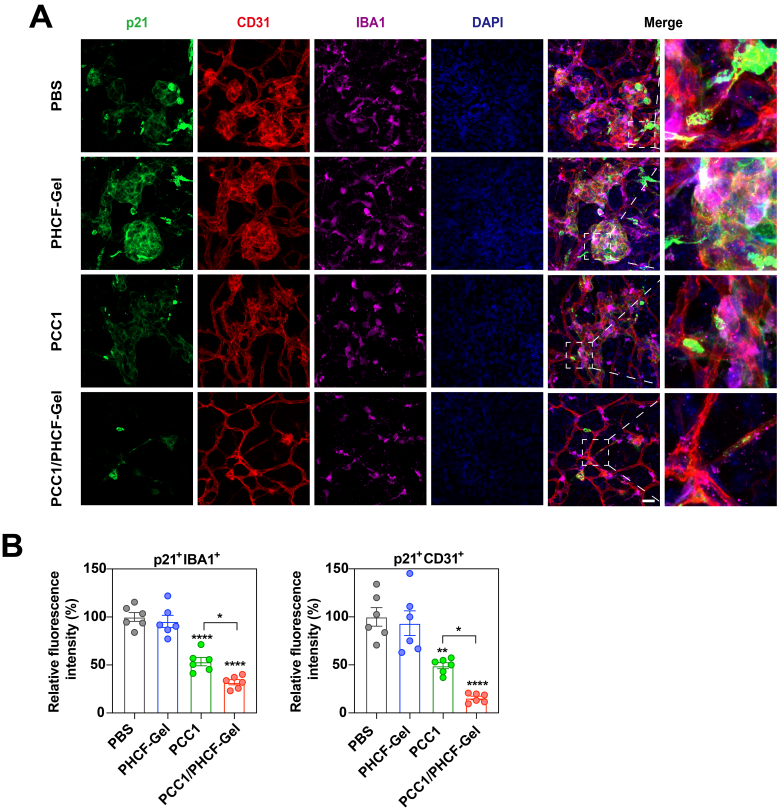


**Figure S17.** (A) Confocal images of retinal flat mounts showed robust staining of senescence markers p21 (green) colabeled with endothelial cells (CD31, red), microglia (IBA1, purple), and nuclei (DAPI, blue). Representative images were taken from P17 OIR mouse retinas 2 days after intravitreal injection with PBS, aflibercept, PHCF-Gel, PCC1, and PCC1/PHCF-Gel. (B) Quantitative analysis of the relative mean fluorescence intensity (n = 6, mean ± SEM). The p-values were calculated using one-way ANOVA with Tukey’s post-test. *p < 0.05, **p < 0.01, ****p < 0.0001; ns, not significance. Scale bar, 20 μm.


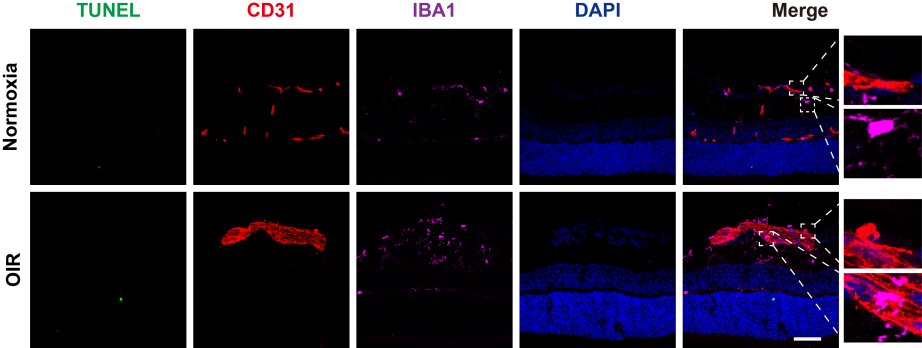


**Figure S18.** Representative confocal images illustrate the apoptotic marker TUNEL (green), endothelial marker CD31 (red), the microglial marker IBA1 (purple), and nuclei (DAPI, blue) in normoxia and OIR mice in vivo. Scale bar, 50 μm.


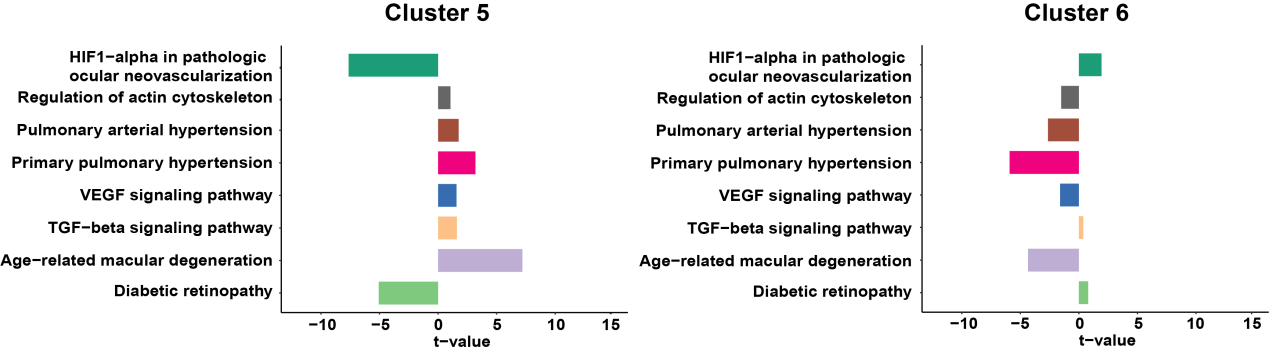


**Figure S19.** The GSVA results of endothelial cell-specific pathways for clusters 5 and 6 in the PBS-treated group.


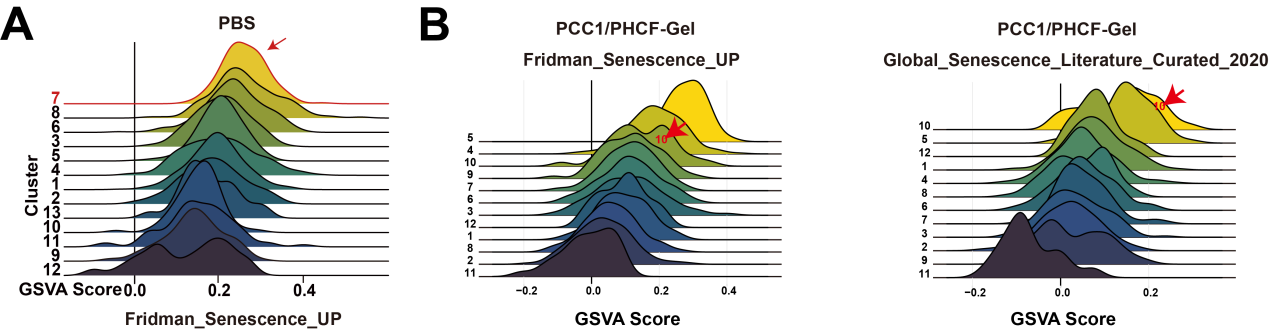


**Figure S20.** (A) Ridge plots showing senescence score distributions in PBS-treated endothelial clusters, calculated using the Fridman_Senescence_UP gene set. The arrow denotes cluster 7. (B) Ridge plots illustrating senescence score distributions across PCC1/PHCF-Gel treated endothelial clusters using Fridman_Senescence_UP and Global_Senescence_Literature_Curated_2020 signatures. The arrow denotes cluster 10.


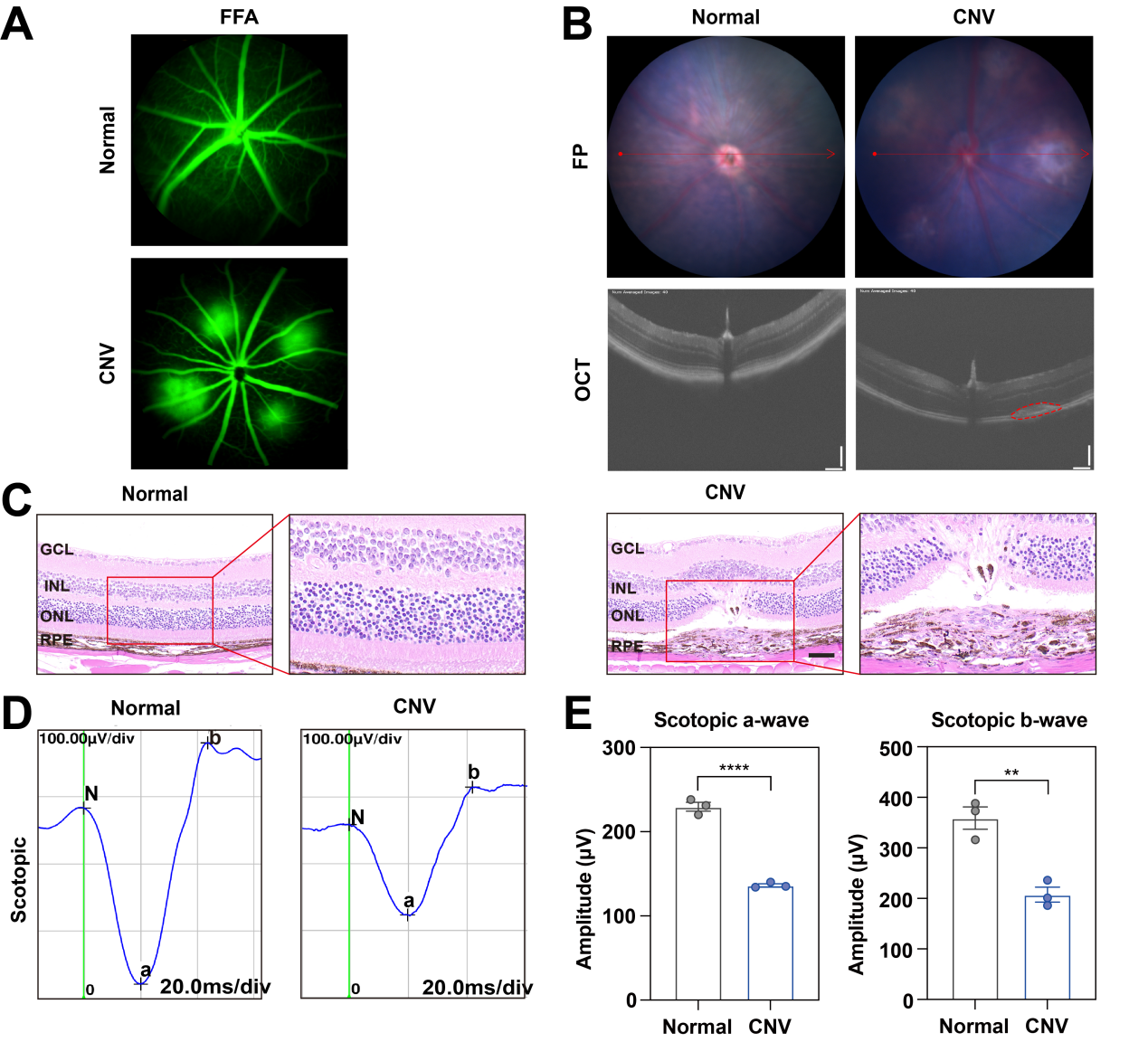


**Figure S21.** (A, B) Representative images of FFA, FP, and OCT of normal mice and laser-induced CNV mice are illustrated, depicting choroidal neovascular lesions observed 3 days after laser-induced injury. (C) Representative H&E staining images of the normal mice and those with laser-induced CNV (GC, ganglion cell layer; INL, inner nuclear layer; ONL, outer nuclear layer; and RPE, retinal pigmented epithelium layer). (D) Representative ERG wave responses of normal mice and laser-induced CNV mice under scotopic conditions, along with quantification of these waves (E). Data are presented as the mean ± SEM (n = 3). The p-values were calculated using Student’s t-test. **p < 0.01, ****p < 0.0001. Scale bars, 100 μm in (B) and 50 μm in (C).

**SUPPORTING TABLE**

| **Table S1. Primary antibodies used in IF analysis** | | | |
| --- | --- | --- | --- |
| **Antibodies** | **Dilution** | **Animal** | **Vendor** |
| γH2AX | 1:200 | Rabbit | Cell Signaling Technologies |
| p21 | 1:200 | Mouse | Santa Cruz Biotechnology |
| p16^INK4a^ | 1:200 | Rabbit | Abcam |
| p53 | 1:200 | Mouse | Cell Signaling Technologies |
| CD31 | 1:200 | Goat | R&D Systems |
| IBA1 | 1:500 | Guinea pig | Synaptic Systems |
| Cxcr4 | 1:100 | Rabbit | Abcam |
| Ifitm3 | 1:100 | Rabbit | Abcam |

**Table S2 Top 10 marker genes**

| **Endothelial** | **Microglia** |
| --- | --- |
| Cxcr4 | MgPVtn |
| Egln3 | Ndufa412 |
| S1c16a3 | Plac9 |
| Tnfrsf9 | S100a6 |
| Kcnq4 | S100a11 |
| Rnd3 | Ifitm3 |
| Ttc9 | Serpinh1 |
| Kdelr3 | Gng11 |
| Lrig1 | Cryab |
| Gpx3 | Crip1 |

**SUPPORTING VIDEOS**

**Videos S1.** Injectable PCC1/PHCF-Gel instantly forms a gel inside the rabbit’s eye after being injected.

**Videos S2.** Characteristics of the rabbit's eye injected with PBS.
